# A monolithic single-chip point-of-care platform for metabolomic prostate cancer detection

**DOI:** 10.1038/s41378-021-00243-4

**Published:** 2021-03-05

**Authors:** Valerio F. Annese, Samadhan B. Patil, Chunxiao Hu, Christos Giagkoulovits, Mohammed A. Al-Rawhani, James Grant, Martin Macleod, David J. Clayton, Liam M. Heaney, Ronan Daly, Claudio Accarino, Yash D. Shah, Boon C. Cheah, James Beeley, Thomas R. Jeffry Evans, Robert Jones, Michael P. Barrett, David R. S. Cumming

**Affiliations:** 1 Electronics and Nanoscale Engineering, James Watt School of Engineering, University of Glasgow, Glasgow, G12 8QQ UK; 2 Beatson West of Scotland Cancer Centre, Glasgow, G12 0YN UK; 3 School of Science and Technology, Nottingham Trent University, Nottingham, NG11 8NF UK; 4 School of Sport, Exercise & Health Sciences, Loughborough University, Loughborough, LE11 3TU UK; 5 Glasgow Polyomics, College of Medical, Veterinary and Life Sciences, University of Glasgow, Glasgow, G61 1BD UK; 6 Institute of Cancer Sciences, Beatson West of Scotland Cancer Centre, University of Glasgow, Glasgow, G12 0YN UK; 7 Institute of Infection, Immunity and Inflammation, University of Glasgow, Glasgow, G12 8TA UK

**Keywords:** Electrical and electronic engineering, Optical materials and structures, Nanoscience and technology, Optical sensors

## Abstract

There is a global unmet need for rapid and cost-effective prognostic and diagnostic tools that can be used at the bedside or in the doctor’s office to reduce the impact of serious disease. Many cancers are diagnosed late, leading to costly treatment and reduced life expectancy. With prostate cancer, the absence of a reliable test has inhibited the adoption of screening programs. We report a microelectronic point-of-care metabolite biomarker measurement platform and use it for prostate cancer detection. The platform, using an array of photodetectors configured to operate with targeted, multiplexed, colorimetric assays confined in monolithically integrated passive microfluidic channels, completes a combined assay of 4 metabolites in a drop of human plasma in under 2 min. A preliminary clinical study using l-amino acids, glutamate, choline, and sarcosine was used to train a cross-validated random forest algorithm. The system demonstrated sensitivity to prostate cancer of 94% with a specificity of 70% and an area under the curve of 0.78. The technology can implement many similar assay panels and hence has the potential to revolutionize low-cost, rapid, point-of-care testing.

## Introduction

One in two people will develop cancer at some point in their lifetime^1^. The World Health Organization estimates that cancer is the second leading cause of death globally, claiming 9.6 million lives in 2018 alone^2^. Cancer incidence is expected to rise by more than 40% in the next 15 years as the population ages^3–5^. With an estimated 1.3 million cases and 0.4 million deaths worldwide in 2018, prostate cancer (PCa) is the most commonly diagnosed cancer in developed countries and the sixth leading cause of cancer deaths in men^5–7^. Although the frequency and survival rate vary considerably for PCa, there is consistent evidence that patients diagnosed at an early stage are more likely to survive^2,3^. Early diagnosis makes localized treatments, including prostatectomy and radiotherapy, possible; hence, the 5-year survival rate is nearly 100%^3,8^. Nevertheless, the survival rate drops to 34% when a tumor is diagnosed in a late metastatic stage^8^.

Despite this, a robust and effective PCa screening program is not available today^9,10^. The widely used prostate-specific antigen (PSA) test, which is the current standard blood test for PCa diagnosis, has been found to be unreliable; fewer than one in three men with an elevated PSA will have PCa^11^, and the test misses ~15% of tumors^12^. The high false-positive rate of the PSA test can lead to unnecessary medical procedures such as digital rectal examination, MRI, and biopsy. In addition to being painful, invasive, and having the potential to cause complications, PSA downstream tests can be expensive^13^, accounting for more than 70% of the medical costs associated with PCa screening. While the use of the PSA test alone is problematic^14^, it has also been suggested that PSA could still be a valuable complement to new and emerging tests such as the one we propose^15^.

While numerous microtechnologies have been proposed to improve and miniaturize PSA tests^16–18^, an independent alternative is sought. One such alternative is to use a panel of metabolite markers that, when taken together, can be analyzed to yield a sensitive and specific test^19–27^. The creation of a panel-based test requires a technological platform that is capable of making multiple simultaneous measurements, ideally in a point-of-care (POC) format that lends itself to regular screening and monitoring that has been shown to be beneficial^28^. Here, we propose the use of a microelectronic test platform based on complementary metal-oxide silicon (CMOS) that underpins all integrated circuit technology. CMOS has the potential to revolutionize multimetabolite marker panel measurements for many diseases, including PCa. Chips with integrated sensors and readouts have been successfully used for single measurements, such as glucose^29^, targeted DNA sequences^30^ and intracellular transmembrane potentials^31^; multiple identical measurements, e.g., genome sequencing^32^; and multianalyte measurements using partitions over a sensing area^33^.

Studies to make devices using micromachining^34^, additive manufacturing^35^, and replica molding^36,37^ have been carried out, but to date, none of these has proven capable of meeting the multimetabolite measurement challenge that must be addressed to build a POC marker panel system. Current methods for CMOS/microfluidic integration and packaging are complex and costly^38^. In addition to building a physical device architecture, solutions are also required for microchannel functionalization, reagent stability^39^, and minimizing crosstalk^40^. Finally, these systems should work with minimal sample preprocessing^39^.

We have overcome these barriers by monolithically integrating a passive microfluidic system onto a CMOS sensor chip to measure multiple metabolites directly from a single droplet of plasma. To do this, it was necessary to control the material dimensions to ensure consistent optical measurements were possible, to control the surface chemistry, hence hydrophilicity, of the channels to ensure passive sampling occurred, and to introduce multiple channel biochemical functionalization on the same chip. In this work, we focused on PCa to demonstrate a new technology with the capability for wide-ranging application and impact.

As described in the Supplementary Information, we selected a panel of 4 metabolites made up of total l-amino acids (LAA), glutamate, choline, and sarcosine. After calibration, the platform was used in preliminary clinical trials with human plasma from 10 healthy men and 16 men diagnosed with PCa. Metabolite profiles were used to train a random forest classifier algorithm. The classifier was shown to have a cross-validated sensitivity of 94% and a specificity of 70% when discriminating between samples from patients with and without PCa, improving upon the current PSA-based clinical standard in the population that we studied.

## The platform

**Fig. 1:**
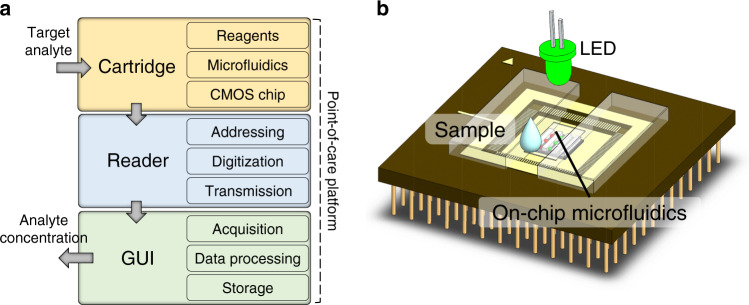
Platform architecture and cartridge. **a** Schematic architecture of the platform showing the cartridge that needs only a drop of sample to perform a measurement, the reader, and the computing device for use as a GUI. **b** A sketch of the multiple measurement cartridge device used in this work with a CMOS chip, passive microfluidics, a chip package, and an optical assembly.

The POC platform is made up of three units (Fig. 1): the disposable chip cartridge, the reader, and the GUI. The apparatus performs colorimetric quantification of a chosen metabolite panel.

### Metabolite panel for PCa

Cancer cells experience rapid proliferation, and their metabolism diverges from healthy cells, giving rise to changes that can be reflected in global measures of the human metabolome^41–43^. Cancer-related metabolites accumulate in human body fluids, and their levels can act as indicators or biomarkers to identify or monitor the disease^44,45^. Many blood metabolites have been found to be linked to PCa^19–27,44–48^. Among them, the progression of cancer is associated with the modification of specific transporters, namely, large amino acid transporters 1 and 3 (LAT1 and LAT3)^19^, which can yield an alteration of the blood LAA profile^20,23,24,46,47^. Cancer cells have been shown to have an upregulated glutamine-glutamate energy cycle; hence, glutamate represents an excellent candidate marker for PCa^20,47,48^. The modification of choline levels, arising from alterations of the enzyme choline kinase-α and the CHT1 choline transporter, has been detected in connection with PCa^21,22^. Finally, sarcosine has also been linked, albeit variably, to PCa for diagnosis^24^, malignancy assessment^23–25^, and staging^23,24,26,27^. While the evidence that sarcosine is a useful marker for PCa is debated^49,50^, we elected to add it to the present study. A more detailed review of metabolomics for PCa and our panel selection is provided in the Supplementary Information.

### Detection strategy

The platform used in this study was developed to quantify the aforementioned candidate metabolic biomarkers using colorimetry. Biological reagents were selected to produce a measurable light absorbance change at a specific wavelength after the interaction with the target metabolite. The initial rate of the reaction is linked to the initial concentration of the metabolite by the Michaelis–Menten model^51^. For the colorimetric determination of LAA, glutamate, choline, and sarcosine, a two-stage reaction process was used to conduct measurements. In the first reaction step, a substrate-specific enzyme was used to produce H_2_O_2_; we used LAA oxidase (LAAOX E.C.−1.4.3.2), glutamate oxidase (GLOX E.C.−1.4.3.11), choline oxidase (CHOX E.C.−1.1.3.17), and sarcosine oxidase (SAOX E.C.−1.5.3.1). The H_2_O_2_ produced was in turn monitored by a colorimetric probe that changed its absorbance properties depending on the H_2_O_2_ concentration. Phenol and 4-aminoantipyrine (4-AAP) were used in this work. In the presence of the catalyzing enzyme peroxidase (HRP), phenol and 4-AAP react with H_2_O_2_, producing quinone imine, which has higher light absorbance in the range of 400–600 nm. The absorbance is linked to the rate of the reaction by the Beer–Lambert law^52^. Detection of an electronic signal was performed using the platform’s array of photodiodes (PDs) to measure the colorimetric reaction.

### The cartridge

**Fig. 2:**
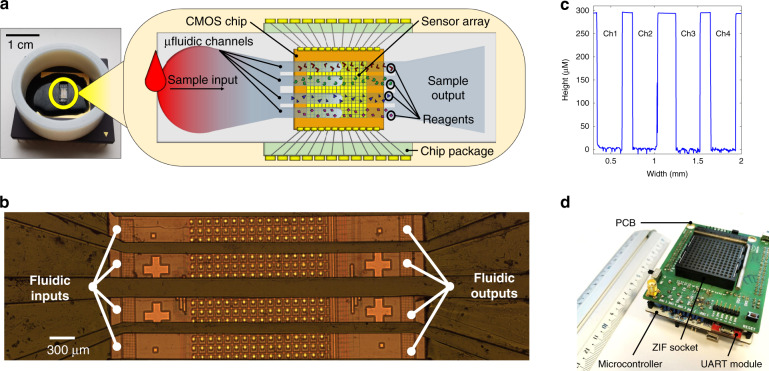
Cartridge and reader. **a** The cartridge (left) and schematic diagram of its main components (right). **b** Micrograph of the microfluidics fabricated on the chip’s sensitive area. **c** Profile of the microstructure built on the sensitive area measured with a Bruker Contour GT-X 3D Optical Profiler (**d**). The handheld reader removed from its enclosure.

The cartridge was made using a ceramic 120 pin grid array (PGA) chip package, a custom complementary metal-oxide-semiconductor (CMOS) chip, a microfluidic capillary network fabricated directly on the chip, and biological reagents. A schematic representation of the main components embedded in the cartridge is shown in Fig. 2a.

The CMOS chip was fabricated using a commercially available 350-nm high-voltage 4-metal process provided by austriamicrosystems (AMS). The chip integrates a 16 × 16 array, or frame, of multisensing elements. Each multisensing element comprises a PD, an ISFET, and a single-photon avalanche diode (SPAD)^53,54^. Only the PDs were used in this work. Each multisensing element is 100 × 100 µm in size, leading to a total sensitive area of 1.6 × 1.6 mm. The size of the entire CMOS chip is 3.4 × 3.6 mm. The CMOS chip was wire-bonded into the PGA.

On top of the sensing area, a passive microfluidic network was monolithically integrated, providing physical separation for parallel testing so that more than one metabolite could be measured at the same time. The walls of the microstructure were fabricated on top of the CMOS chip using a biocompatible black epoxy resin (302-3M 1LB by Epoxy Technology Inc.) using a combination of soft lithography and injection molding. A plain polydimethylsiloxane (PDMS) slab coated with polyvinyl alcohol (PVA)^55^ was bonded onto the epoxy-based structure by plasma oxidation to enclose the channels from the top. A detailed description of the microfluidic integration is presented in the Materials and Methods section of this paper. As shown in Fig. 2b, c, the microchannel height, width, and length were ~291.95 ± 6.44 µm, 300.87 ± 0.86 µm and 4.0 ± 0.1 mm, respectively. A liquid sample (see “Materials and methods” section) was introduced to the cartridge using a pipette (Finntip^TM^ F2 by ThermoFisher). Once on the cartridge, the sample under test was divided into four identical microfluidic channels that physically confined the reactions in each channel.

Two types of experiments were conducted: a series to measure each metabolite one-by-one to assess the validity of the proposed panel and a second to make four measurements in parallel and demonstrate the potential of the platform. When testing a single metabolite, the sample and the bioreagent were mixed in the liquid state immediately before loading onto the chip to perform the test. Thus, both the sample and bioreagents are introduced to the system at the same time. When testing multiple metabolites in parallel, the channels were individually functionalized with the different bioreagents required to detect each metabolite. Biological reagents were preloaded into the microchannels by manual pipetting and then dried. The procedure entrapped and isolated the solid materials in their respective microchannels.

### Reader and graphical user interface

The cartridge plugs into the reader using a zero-insertion force (ZIF) socket. The reader is 8.5 × 7.5 × 4.0 cm and weighs 150 g. The reader provides functionality for sensor multiplexing and addressing, data digitization and transmission to a personal computing device via a USB link. The reader is based around an STM32F334R8T6 microcontroller on an ST Nucleo F334R8 board (Fig. 2d) that is programmed before use with custom firmware. Data are digitized using the embedded 12-bit analog to digital converter with an average rate of 36 frames per second. The reader is powered by the USB link (5 V), which in turn powers the cartridge (3.3 V). The reader also has an LED (*λ* = 490 nm, FWHB = 20 nm). Using a lens (AC254-035-A-ML BBAR Coated, *f* = 35 mm lens from Thorlabs), the LED illuminates the sensing area of the cartridge with collimated light. The GUI, based on custom software and running on a personal laptop (HP EliteBook i7-8650u 16 GB), interfaces with the reader (via USB) and performs data acquisition, display, analysis, and storage. The results can also be uploaded onto a cloud. The GUI also performs offline signal processing. Additional details (Supplementary Fig. 4) of the GUI are reported in the Supplementary Information.

## Results

The platform was tested and characterized using human plasma samples modified with known concentrations of metabolites so that calibration curves could be generated. Subsequently, a preliminary clinical study for PCa was performed using ten samples from healthy men not known to have PCa (non-PCa group) and sixteen samples from men affected by PCa.

### Calibration

**Fig. 3:**
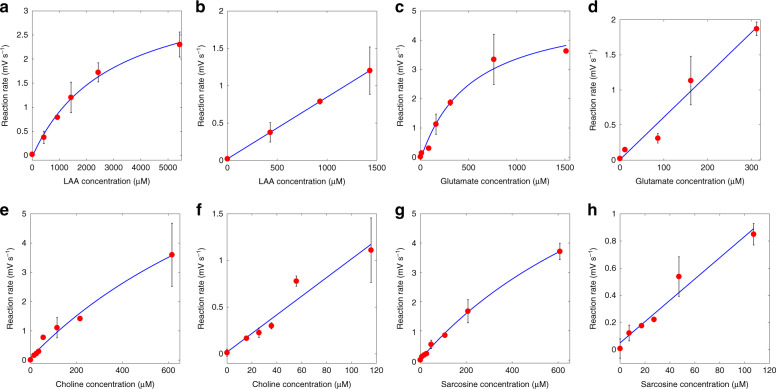
Calibration curves. Calibration curves obtained by fitting the data with Michaelis–Menten and linear models for **a**, **b** LAA, **c**, **d** glutamate, **e**, **f** choline, and **g**, **h** sarcosine in human plasma.

Calibration curves for LAA, glutamate, choline, and sarcosine in human plasma are shown in Fig. 3. The complete characterization of the platform for the analytes of interest is presented in Table 1. At least six data points were used to obtain the calibration curves for each metabolite. Each data point was obtained as the average over three replicates (see “Materials and methods” section). Kinetic constants were estimated by fitting data to the Michaelis–Menten model. The *K*_m_ results for all of the metabolites were in line with the values reported in the literature^56^. For all the metabolites, the goodness of fit with the Michaelis–Menten model was satisfactory with *R*^2^ values ≥0.97. For substrate concentrations lower than *K*_m_, the data were also fitted using a linear model. For all the metabolites, high linearity was observed in the concentration range of interest (*R*^2^ ≥ 0.93). The linear ranges were in line with the physiological ranges. Typical standard deviations for the measurements in the linear range were found to be 16–20%.

**Table 1:** Platform characterization using human plasma.

	LAA	Glutamate	Choline	Sarcosine
Physiological range	2–3.5 mM^68^	40–150 µM^47^	7–20 µM^69^	0–20 µM^50^
Test range	0–5.4 mM	0–1500 µM	0–600 µM	0–600 µM
Model	$y = \frac{{V_{{\mathrm{max}}}x}}{{K_{\mathrm{m}} + x}} + c$			
*V*_max_ (mV s^−1^)	3.63 ± 0.51	5.28 ± 0.93	11.34 ± 6.9	11.03 ± 2.1
*c* (mV s^−^^1^)	−0.032 ± 0.126	−0.087 ± 0.265	0.082 ± 0.254	0.027 ± 0.04
*K*_m_ (µM)	2866 ± 1008.2	529.7 ± 269.5	1382 ± 210.7	1209 ± 335.7
RMSE	0.086	0.266	0.169	0.062
*R*^2^	0.994	0.979	0.985	0.998
Linear model	*Y* = *S* ⋅ *x* + *C*			
Linear range (µM)^a^	0–1500	0–320	0–120	0–120
Sensitivity (*S*) (mV s^−1^ mM^−1^)	0.83 ± 0.002	6.06 ± 1.01	9.98 ± 1.79	7.84 ± 1.12
Baseline (mV s^−1^)	0.020 ± 15·10^–4^	0.003 ± 0.163	0.019 ± 0.1	0.050 ± 0.056
RMSE (linear)	8.6⋅10^−04^	0.159	0.116	0.070
*R*^2^ (linear)	0.999	0.969	0.939	0.961
Average relative std. dev. (linear)^b^	18.3%	17.2%	16.4%	19.2%
Negative control (µV s^−1^)^c^	5.0 ± 2.7			
LOD (µM)^d^	11.1	1.4	1.7	1.4
LOQ (µM)^d^	25.5	3.3	3.9	3.5
Drift (dark/source on) (µV s^−1^)	1.4 ± 1.0/0.9 ± 1.0			
Avg. steady state (dark/source on) (V)	0.486 ± 0.003/1.730 ± 0.031			

^a^Linear range is here defined as the measurement range where the linear model had *R*^2^ > 0.9.

^b^Average of the standard deviation of the measurements in the linear range.

^c^Average over 24 measurements.

^d^Converted from mV s^−1^ to µM using the Michaelis–Menten model.

### Limit of detection and limit of quantification

The limit of detection (LOD) and limit of quantification (LOQ) were quantified using the “International Union of Pure and Applied Chemistry” (IUPAC) definition^57^. The average (*µ*_c_) and standard deviation (*δ*_c_) of the initial reaction rate for negative controls (common to all the assays) were found to be 5 and 2.7 µV s^−1^, respectively. Consequently, the LOD (*µ*_c_ + 3.3·*δ*_c_) and LOQ (*µ*_c_ + 10·*δ*_c_) were 0.014 and 0.032 mV s^−1^, respectively. LOD and LOQ expressed in mV s^−1^ were then converted to µM by using the estimated Michaelis–Menten curve for each metabolite. Thus, the LODs for LAA, glutamate, choline, and sarcosine were 11.1, 1.4, 1.7, and 1.4 µM, respectively. Similarly, the LOQ values for the metabolites in the same order were 25.5, 3.3, 3.9, and 3.5 µM.

### Clinical study

For each sample, the plasma concentrations of LAA, glutamate, choline, and sarcosine were quantified using the experimental platform. The procedures and methods are described in the “Materials and methods” section of this paper.

The average concentration of a metabolite for all samples, including the non-PCa and PCa subjects, is referred to as the grand average. A grand average was calculated for each of the four metabolites we measured. The grand averages were 2421 ± 952 µM for LAA, 53.7 ± 26.4 µM for glutamate, 11.7 ± 7.0 µM for choline, and 10.6 ± 6.0 µM for sarcosine.

The average values measured for each metabolite for the non-PCa and PCa groups were also calculated. For the non-PCa samples, the average concentrations were 1984 ± 527 µM for LAA, 40.2 ± 11.2 µM for glutamate, 10.0 ± 4.1 µM for choline, and 11.5 ± 4.3 µM for sarcosine. The average concentrations of LAA, glutamate, choline and sarcosine in the PCa group were 2694 ± 1052 µM, 62.2 ± 29.5 µM, 13.4 ± 7.9 µM, and 10.0 ± 6.9 µM, respectively.

**Fig. 4:**
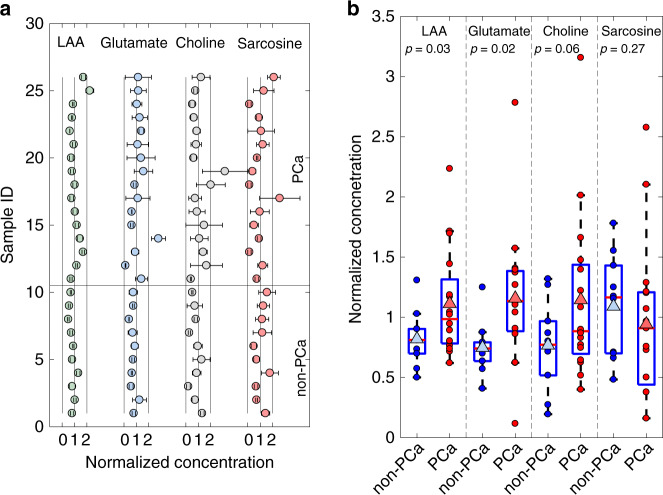
Results of the clinical study. **a** Metabolite quantification in the non-PCa (samples 1–10) and PCa groups (samples 11–26). The concentration of the metabolic biomarkers was normalized to the grand average. **b** Univariate analysis and box plots for the non-PCa group vs the PCa group. Blue markers indicate non-PCa measurements. Red markers indicate PCa measurements. The triangular markers indicate the average values in the non-PCa and PCa groups.

LAA, glutamate, and choline levels were increased in the PCa group compared with the non-PCa group. There was no relevant cross-correlation between different metabolites, and all cross-correlations were <0.3 (Supplementary Information). These data are summarized in Table 2 and presented in Fig. 4.

**Table 2:** Clinical study results in the control and cancer groups.

	LAA	Glutamate	Choline	Sarcosine
*Overall data set*				
Grand average ± std. dev. (µM)	2421 ± 952	53.7 ± 26.4	11.7 ± 7.0	10.6 ± 6.0
Grand median (µM)	2072	47.9	10.0	9.9
Range (µM)	1213–5421	6.3–149.5	2.3–36.9	1.7–27.2
Temperature (°C)	27.3 ± 1.0	26.4 ± 1.3	26.3 ± 0.9	25.9 ± 1.2
Humidity (%)	52.6 ± 5.0	49.5 ± 7.8	44.4 ± 9.0	42.2 ± 10.5
*Non-PCa group*				
Non-PCa average ± std. dev. (µM)	1984 ± 527	40.2 ± 11.2	10.0 ± 4.1	11.5 ± 4.3
Non-PCa median (µM)	1966	39.8	9.0	12.3
Range (µM)	1213–3167	21.9–67.1	2.3–15.4	5.1–18.8
*PCa group*				
PCa average ± std. dev. (µM)	2694 ± 1052	62.2 ± 29.5	13.4 ± 7.9	10.0 ± 6.9
PCa median (µM)	2386	61.0	10.4	9.7
Range (µM)	1503–5410	6.3–149.5	4.7–36.9	1.7–27.2
*Univariate analysis*				
PCa/non-PCa (average)	1.36	1.55	1.34	0.87
PCa/non-PCa (median)	1.21	1.53	1.15	0.79
*t*-test (*p*-value)	0.03	0.02	0.06	0.27

### Multivariate analysis

**Fig. 5:**
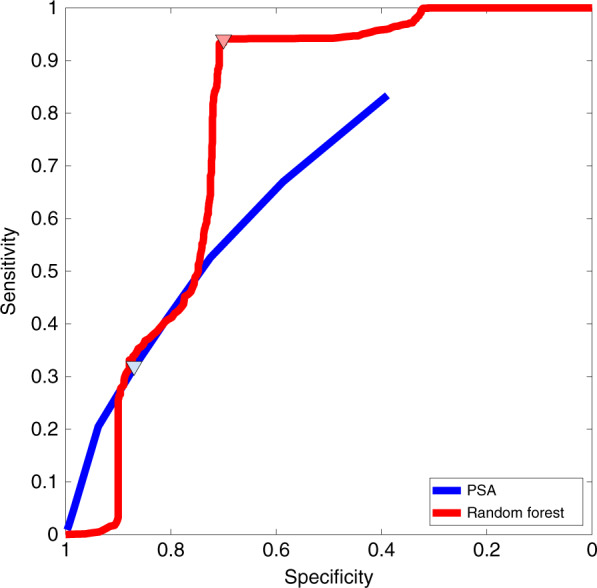
Multivariate analysis. ROC curve for the cross-validated random forest algorithm trained with the measured metabolic profiles of LAA, glutamate, and choline. The ROC curve is compared with that for PSA^61^. Operating points are shown using triangular markers.

To determine the validity of using LAA, glutamate and choline as potential diagnostic markers, the data set was used to train a random forest classification algorithm^58^. The task of the classifier was to provide a binary “negative-or-positive” response to whether a sample was a control (negative) or cancer (positive) sample, using the concentrations of LAA, glutamate and choline as inputs. Using the “randomForest” and “caret” functions in the R software tool, the algorithm was set to use 500 trees and try up to three metabolites at each split. The model was validated using a repeated “tenfold” procedure that was run 100 times^59,60^. In this way, we generated a cross-validated receiver operator characteristic (ROC) curve using the predictions over every iteration. For each iteration, a bootstrap resampling procedure was used. The metrics of the classifier were expressed as an average, and a 95% confidence interval over the distribution was obtained for the 100 independent training and validation iterations. The area under the curve (AUC) was found to be 0.78, with a 95% confidence interval of 0.55–0.99. The ROC curve shows an operating point at a sensitivity of 0.94, with a 95% confidence interval of 0.82–1.00, and a specificity of 0.70, with a 95% confidence interval of 0.40–0.98, as shown in Fig. 5. The diagnostic capability of the classifier can be compared with that of PSA. In clinical practice, the PSA sensitivity and specificity are 0.32 and 0.87, respectively, for a PSA threshold of 3.1 ng mL^−1^ ^61^. PSA yields an AUC of 0.68^61,62^. Our results show that the random forest model based on LAA, glutamate, and choline could substantially reduce the number of false-positive results.

These results show that the platform has the potential to deliver higher diagnostic capability than PSA. We also note that if both the PSA and metabolomic test were to be used together, it would be independently possible to secure both a high sensitivity of up to 94% (metabolites) and high specificity of 87% (PSA) to yield a powerful and highly discriminating diagnostic method.

### Simultaneous measurements

To make a practically useful POC diagnostic tool using the markers LAA, glutamate and choline, it is desirable to perform simultaneous multimetabolite measurements. Simultaneous measurements require reagents to be preloaded into the microfluidic channels. Sarcosine was excluded from these experiments since it was concluded that it was not a useful biomarker for this population.

**Fig. 6:**
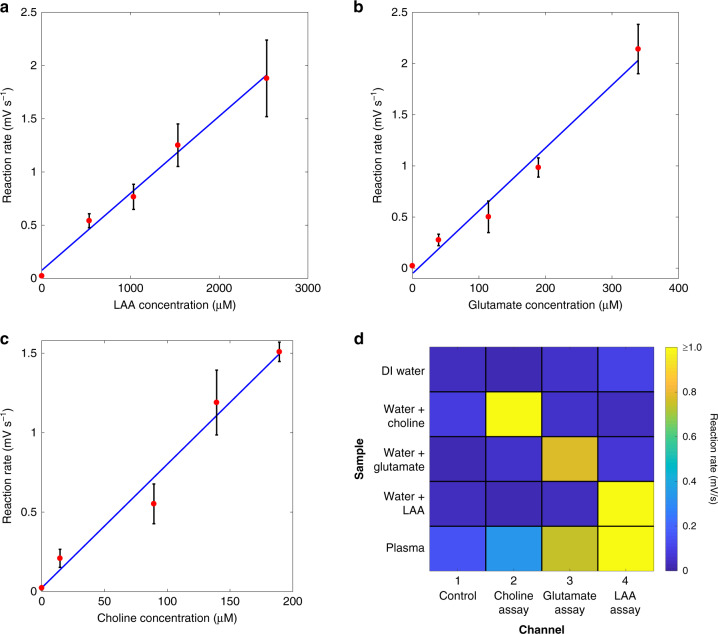
Assays with preloaded reagents. Calibration curves for plasma **a** LAA, **b** glutamate, and **c** choline obtained with reagents preloaded in the microfluidic channels in the dried state. **d** Control experiments to evaluate the potential crosstalk for multimetabolite sensing. Metabolites in plasma have different physiological concentrations. Hence, there is a difference in the absolute value of the reaction rate. The blue sectors in the first four lines indicate that there was very little crosstalk.

The performance of the platform with preloaded dried reagents (see Materials and Methods section) was assessed by obtaining calibration curves for plasma LAA, glutamate, and choline in the microfluidic device. Each metabolite was tested individually in undiluted human plasma spiked with the metabolite to the desired test concentration. The resulting calibration curves are shown in Fig. 6a–c. The results were similar to the calibration curves obtained from using off-chip mixing of the liquid reagents. A comparison between the two test methods is shown in Table 3. The linearity in the physiological range was nearly the same, but a slight loss of sensitivity for LAA and choline when using the dried reagents was observed. The control channel, which contained dried dye and peroxidase only, showed a measurable response when compared to photodiodes with no dried assay material present. The observed control signal was still small compared to the signals observed in the test channels and gave rise to the increase in the calculated LOD and LOQ for all the target metabolites. Under these conditions, the LODs for plasma LAA, glutamate, and choline were 42.9, 6.4, and 3.2 µM, respectively. Similarly, the LOQ values for the metabolites, in the same order, were 129.3, 19.5, and 9.8 µM.

**Table 3:** Comparison of the performance of the platform for the quantification of LAA, glutamate, and choline when using off-chip mixed liquid reagents or preloaded dried reagents.

	LAA		Glutamate		Choline	
Physiological range:	2–3.5 mM^68^		40–150 µM^47^		7–20 µM^69^	

Reagents:	Liquid	Dried	Liquid	Dried	Liquid	Dried
Sensitivity (mV s^−1^ mM^−1^)	0.83 ± 0.002	0.72 ± 0.07	6.06 ± 1.01	6.14 ± 0.87	9.98 ± 1.79	7.78 ± 1.13
Linearity (*R*^2^)	0.999	0.991	0.969	0.977	0.939	0.975
RMSE	0.086	0.076	0.266	0.145	0.169	0.115
Average std. dev. (%)	18.3%	15.7%	17.2%	18.0%	16.4%	17.7%
Negative control (µV s^−1^)	5.0 ± 2.7	23.0 ± 12.5	5.0 ± 2.7	23.0 ± 12.5	5.0 ± 2.7	23.0 ± 12.5
LOD (µM)	11.1	42.9	1.4	6.4	1.7	3.2
LOQ (µM)	25.5	129.3	3.3	19.5	3.9	9.8

To evaluate channel-to-channel independence on the same chip, the four channels in a set of 12 cartridges were filled with dried reagents for LAA, glutamate, choline and a negative control. Using one cartridge at a time, triplicates of each of the following were measured by flowing the sample into the channels: DI water; 250 µM choline in DI water; 250 µM glutamate in DI water; and 2.5 mM LAA in DI water. There was no response to DI water only, and as expected, each functionalized channel only responded to the metabolite for which it had been prepared.

A further triplicate of cartridges was prepared with the three functionalized channels and a control, and in each, an unmodified human plasma sample was introduced to the cartridge. The plasma sample yielded signal rates above the LOQ for LAA, glutamate, and choline measurements. No obvious crosstalk was observed in these experiments, and the results are summarized in Fig. 6d.

**Fig. 7:**
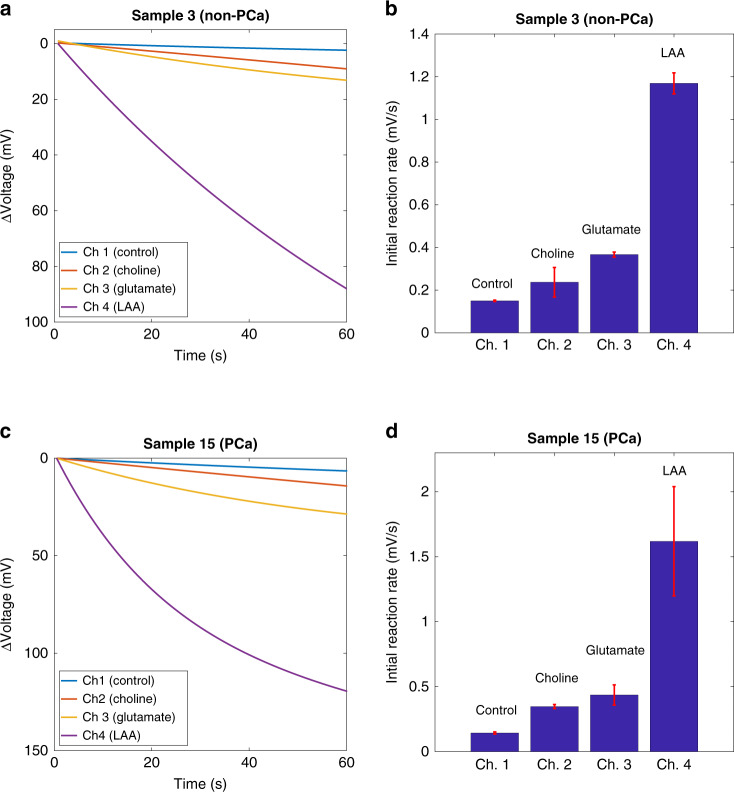
Simultaneous testing with clinical samples. **a** Initial reaction rates in four different microfluidic channels for a non-PCa sample. **b** Output from different microchannels for a non-PCa sample. **c** Initial reaction rates in four different microfluidic channels for a PCa sample. **d** Output from different microchannels for a PCa sample.

A proof of principle clinical validation of the platform for multimetabolite testing using dried reagents in a single cartridge was then conducted using one individual each from the non-PCa and PCa sample groups. Simultaneous readings for different metabolites are shown in Fig. 7. For both clinical samples, the reaction rates were found to be similar to the respective wet assay. The rates obtained with dried and liquid reagents were well correlated with *R*^2^ > 0.91.

The rates obtained with dried reagents for non-PCa and PCa samples were also compared; the rates were consistently higher for the PCa sample, as was the case with the wet assays. This provides a proof of principle that the platform can provide clinically relevant information when testing for the metabolites of interest simultaneously.

## Discussion

We have shown that the method for acquiring data using multiple metabolites can be integrated into a silicon chip-based device capable of making all the measurements simultaneously. Using the device, a preliminary clinical study demonstrated that a model can be created using multiple metabolites to discriminate patients with PCa from normal controls. LAA, glutamate and choline showed a significant correlation in our population, and the data were used to train a random forest classification model. In our study, sarcosine did not show any relevant correlation with PCa; therefore, the data were not used for the classifier. The ROC curve for the new test was found to have an AUC = 0.78 that compared favorably with an AUC = 0.68 for the PSA test. The test ROC curve intersects the PSA ROC curve near the standard threshold with a specificity of ~87% and a sensitivity of 32%. However, the new test’s ROC curve has a significantly greater sensitivity of 94% when operated at a slightly lower specificity of 70%.

The metabolic biomarker panel we present provides a valuable proof-of-concept and can potentially be improved by including additional metabolites. Table 3 in the Supplementary Information presents additional PCa-related blood metabolites that could be quantified using the platform. The availability of data on a larger marker panel will enable a comprehensive analysis of the proposed diagnostic method. Further work with a larger population of subjects leading to a full clinical trial using the diagnostic method proposed in this paper will be necessary to demonstrate that the platform can deliver an effective POC diagnostic tool for PCa.

We envisage a potential scale-up to build a system capable of measuring a whole-person metabolome in a single measurement from a drop of blood. The procedures and methods developed in this work can be optimized to improve the LOD and LOQ and applied to a larger CMOS sensor array to deliver increased multiplexing capabilities. Future work will include developing methods for highly dense measurement multiplexing with low crosstalk. The technology may also take advantage of the computation capabilities of CMOS to create a device capable of not only collecting raw data but also integrating complete machine learning algorithms to yield a highly sensitive general-purpose chip, or chip-family, capable of extracting large amounts of highly specific and individualized data.

## Conclusion

Survival rates for many types of cancer are continuing to improve^63^, but progress towards improving the outcome for men with prostate cancer has been hindered by the need for a reliable test. The lack of such a test has inhibited the introduction of mass screening programs. As a consequence, many instances of cancer are only detected very late, when the possibilities for effective treatment are reduced. The CMOS point-of-care platform presented in this paper has the potential to address this problem by improving the accuracy of a diagnostic test to such an extent that screening will become a more clear-cut choice. Future tests may combine the merits of more than one assay; hence, metabolite measurements could be used in conjunction with a test for PSA. Indeed, progress is also being made to develop POC tests for PSA^16^. We provide proof-of-concept for a POC platform using a CMOS sensor chip with monolithically integrated microfluidics that is capable of performing multiple metabolite tests pertinent to the diagnosis of prostate cancer simultaneously. The system was shown to be capable of detecting diagnostically significant information in the population under test and can be used to improve the current clinical standard. Furthermore, the platform has the potential to be used in a domestic environment and is therefore capable of detecting early changes in candidate biomarkers when measured over a period of time. The technology presented in this article has wide-reaching implications, not only for cancer, as illustrated in Table 1 of the Supplementary Information but for other diseases and personalized medicine. Metabolite marker panels are now described for illnesses including sepsis^64^, acute kidney injury^65^, and cardiovascular disease^66^. We anticipate a future microelectronic platform to exploit the scalable properties of CMOS that will become as commonplace in medicine as the stethoscope and thermometer are today.

## Materials and methods

### Microfluidic design

Microfluidic channels were designed to provide laminar and passive flow. The geometry of the design, composed of straight microchannels with rectangular cross-sections, was chosen to match the layout of the CMOS sensor array. Custom MATLAB simulations were carried out to identify the dimensions of the microchannels needed to yield higher capillary pressure and lower filling time in the laminar flow regime.

### Microfluidics integration

Microfluidics was integrated with the CMOS chip by replica and injection molding in five stages: (1) SU-8 mold fabrication, (2) PDMS mold fabrication, (3) wire bonding, (4) epoxy encapsulation, and (5) channel enclosure.

1. A silicon wafer (4″) was cleaned with IPA, acetone, and DI water; sonicated; dehydrated (10 min, 90 °C); and plasma-oxidized (2 min, 120 W). A first SU-8 3050 layer was spin-coated onto the wafer (30 s, 1000 rpm) and baked (90 min, 90 °C). A second SU-8 3050 layer was similarly spin-coated and baked. The substrate was exposed twice to UV light using a mask aligner (70 s each time, 15 s wait time). Afterward, the sample was baked (10 min, 90 °C), developed using EC solvent (for 28 min), rinsed with IPA, and baked (30 min, 180 °C).
2. The SU-8 mold was silanized by exposure to trichlorosilane (30 min in an evacuated chamber) and placed into a petri dish. PDMS (25 g, 1:14 ratio) was poured onto the mold, degassed (1 h in a vacuum chamber), and cured (2 h, 70 °C). Cured PDMS was released from the SU-8 mold, placed on a clean substrate, cut with a sharp knife, aligned, and temporarily bonded to the CMOS chip using a flip-chip bonder (model 850, Semiconductor Equipment Corp.). The bond strength was increased by heating the two respective part holders for the chip and PDMS (90 °C, 10 min) under constant pressure (5 psi).
3. The CMOS chip with the bonded PDMS mold was glued into the 8.3 × 8.3 mm cavity at the center of a 120 pin ceramic chip pin grid array package using EPO-TEK H74 epoxy (Epoxy Technology Inc.) and wire-bonded (by Hesse and Knipps Bondjet 710).
4. A black epoxy resin (302-3M 1LB, Epoxy Technology Inc.) was injected into the PDMS microstructure and cured (48 h at room temperature). After curing, the PDMS structure was removed from the chip. Because there were no wire bonds on the top and bottom edges of the chip, microchannels were extended in these directions, effectively planarizing the surface. Epoxy also provided encapsulation of the wire bonds.
5. A planar slab of PDMS was cut with a sharp knife (4 × 3 mm), cleaned, exposed to oxygen plasma (1 min, 80 W) and immersed in a PVA solution (1 wt%)^55^. The PVA-modified PDMS slab was permanently bonded to the epoxy microstructure by plasma activation (45 s, 80 W) and baking (15 min, 90 °C).

The approach that was used encapsulated all the water-sensitive electronic components on the cartridge in epoxy. This enabled leakage-free aqueous experiments on the cartridge. A graphical representation of the fabrication is shown in Fig. 3 of the Supplementary Information.

### Reagents

All chemicals required for the assays were purchased from Sigma-Aldrich unless otherwise specified. A reagent solution per target metabolite was prepared immediately before the experiment. Assay formulations were optimized by experimentation. All the reagents were prepared in DI water.

For LAA testing, 6.7 µL of LAAOX (10 U mL^−1^), 6.7 µL of HRP (150 U mL^−1^), 3.3 µL of phenol (44.5 mM) and 3.3 µL of 4-aminoantipyrine (4-AAP, 10.5 mM) were mixed. The reagent solution for glutamate was prepared by mixing 6.7 µL of GLOX (4 U mL^−1^), 6.7 µL of HRP (150 U mL^−1^), 3.3 µL of phenol (44.5 mM), and 3.3 µL of 4-AAP (10.5 mM). For choline testing, 6.7 µL of CHOX (150 U mL^−1^), 6.7 µL of HRP (300 U mL^−1^), 3.3 µL of phenol (44.5 mM), and 3.3 µL of 4-AAP (10.5 mM) were mixed. The reagent solution for sarcosine was prepared by mixing 6.7 µL of SAOX (200 U mL^−1^), 6.7 µL of HRP (300 U mL^−1^), 3.3 µL of phenol (44.5 mM), and 3.3 µL of 4-AAP (10.5 mM). The reagent solution for the negative control was prepared by mixing 6.7 µL of DI water, 6.7 µL of HRP (300 U mL^−1^), 3.3 µL of phenol (44.5 mM), and 3.3 µL of 4-AAP (10.5 mM).

### Experimental setup

To ensure consistent results, a rigid test setup was built. All optomechanical components were purchased from Thorlabs. The reader of the platform was secured to an optical aluminum breadboard (15 cm × 10 cm × 1.2 cm) with the ZIF socket facing up. Cartridges were inserted into the ZIF socket. The sensitive area of the CMOS chip was parallel to the optical breadboard and facing up. Using optomechanics, a 3 mW LED (*λ* = 490 nm, FWHM = 20 nm) powered using a power supply unit (HP E3631A) was used to uniformly illuminate the sensing area on the CMOS chip with a collimating lens (AC254-035-A-ML BBAR coating *f* = 35 mm). The height and positioning of the LED and lens were adjusted so that the sensitive area of the chip received perpendicular collimated light. The equipment that was mounted onto the optical breadboard was enclosed in a box and covered with a nylon/polyurethane blackout cloth. The cloth was essential to ensure that the experiments were performed in a dark environment. A small opening was left for connecting wires and allowed sample delivery to the chip. The reader, enclosed in the dark environment, was connected to an external laptop (HP EliteBook i7-8650u 16 GB) using a USB cable. The MATLAB-based user interface, running on the laptop, was used to control data acquisition. Data were recorded with an average frame rate of 36.5 fps and a resolution of 12 bits. Typically, the duration of a single experiment was 5 min (~10,950 frames). Environmental temperature and humidity were also monitored during testing using a Texas Instrument module (HDC 1080EVM).

### Cartridge reusage

Although the cartridge could in principle be a single-use disposable device, because of limited resources, in this work, cartridges were cleaned and reused. A cleaning procedure after every measurement was used to avoid cross-contamination. The cleaning process involved a sequential rinse in DI water, IPA, and then ethanol, and nitrogen was used to blow it dry. For the clinical samples, an additional first rinse step with a dilute piranha solution was performed. The dilute piranha solution was prepared using 10:3:1 DI water:18 M sulfuric acid:30% hydrogen peroxide. Cleaning the cartridge with dilute piranha was kept to a minimum since the etchant attacked the epoxy microchannels. Cross-contamination reduction was also achieved by optimizing the testing sequence. In particular, one or more negative controls were performed before any measurements.

### Calibration

For calibration with liquid reagents, one human plasma sample was purchased from Sigma-Aldrich and reconstituted according to the manufacturer’s instructions. Subsequently, it was modified by adding known quantities of analytes of interest. Additional concentrations did not take into account the unknown endogenous level of the substrate of interest in the sample. The endogenous concentration was estimated by using the method for substrate measurement described in the signal processing section. Twenty microliters of reagent solution was mixed off-chip with 20 µL of sample and introduced into the cartridge within a few seconds.

For calibration with dried reagents, human plasma was purchased, reconstituted, and modified using the same procedure described for calibration with liquid reagents. The same reagents were preloaded in all four microchannels in a single cartridge. Thirty microliters of sample was introduced to the cartridge without any further dilution.

For both configurations, metabolites were tested individually. Each cartridge had four microchannels; therefore, each measurement yielded four reaction rates. The method employed for sample delivery was reliable and repeatable; therefore, air bubbles or fluidic failures did not pose a problem for the majority of the experiments. However, in a small number of instances, unexpected behavior was observed, and the data were excluded. The four reaction rates were averaged^67^, and the small number of anomalies that occurred was mitigated using triplicate measurements from each cartridge. Each cartridge was functionalized with dried reagents prior to each new measurement. The errors were expressed using the standard deviation of these data.

### Non-PCa samples

Ten samples of human plasma from healthy people, herein referred to as “non-PCa”, were commercially sourced from Cambridge Bioscience. The exclusively adult male non-PCa donors were age 34 ± 10 years. The ethnicity of the group was diversified. The samples were tested for the most common infectious diseases, and all gave negative results. Approximately 10 mL of fresh blood samples were collected from subjects in various research centers in England, mixed with 10 mg of K_2_EDTA anticoagulant and centrifuged. The resulting 4-mL plasma samples were frozen at −80 °C and shipped under dry ice. After collection, plasma samples were aliquoted and stored at −80 °C. No additional freeze and thaw cycle was performed.

### PCa samples

Sixteen samples of human plasma from people diagnosed with PCa, herein referred to as the “PCa group”, were sourced from the Beatson West of Scotland Cancer Centre, Glasgow, UK, using an ethically approved sample collection protocol. Donors were adults already diagnosed with PCa. All patients were under treatment. Approximately 10 mL of blood samples was collected at the cancer center, mixed with 10 mg of K_2_EDTA anticoagulant, and centrifuged, and the resulting plasma samples were frozen at −80 °C. Samples were transported in dry ice. Afterward, plasma samples were aliquoted and stored at −80 °C. No additional freeze and thaw cycle was performed. A copy of the ethical approval letter and consent forms from the donors are available upon request.

### Preliminary clinical study

The non-PCa and PCa groups were tested for the four metabolites of interest for PCa diagnosis. Chronologically, the non-PCa group was tested before the PCa group. For convenience numbered sample IDs were assigned. Samples with IDs from 1 to 10 belong to the non-PCa group. Samples with sample IDs from 11 to 26 belong to the PCa group.

Within each group, metabolites were measured one-by-one in the following order: LAA, glutamate, choline, and sarcosine. Experiments were performed with wet reagents. Twenty microliters of reagent solution was off-chip mixed with 20 µL of clinical sample and introduced onto the platform within a few seconds. For each sample and metabolite, the negative control (background) was first assessed. Then, the assay was performed. Finally, positive controls A and B were tested. Additional concentrations of positive control A for LAA, glutamate, choline, and sarcosine were [A] = 500 µM, 100 µM, 100 µM, and 100 µM, respectively. The additional concentration for positive control B was [B] = 2[A]. Controls were obtained using single measurements. The assay was repeated three times using the same cartridge. Averages and standard deviations were obtained for the three measurements.

### Microchannel functionalization with dried reagents

To functionalize the microchannels with dried assay material, reagent solutions for the control and for LAA, glutamate, and choline assays were first prepared as described above. One microliter of each reagent solution was preloaded into the required microchannel by manual pipetting. Ultralong microloader pipette tips (Eppendorf) with an outer diameter of 100 µm were used under a microscope. The pipette tips made it possible to dispense the reagent directly into single microchannels. To avoid contamination of the shared input fluidic region, reagent solutions were inserted from the fluidic output-end of the channels. After the deposition of the reagent solutions, the cartridge was dried for 1 h at room temperature in a vacuum chamber. The control channel was preloaded with a reagent solution containing HRP, phenol, and 4-AAP. The presence of dried reagents on the chip slightly increased the light absorbance of the platform after settling. To compensate, the intensity of the light from the LED was increased to keep the PDs at the same operating point with respect to the unfunctionalized microchannels.

Reagents were rehydrated when the sample was introduced into the microchannels. Based on visual inspection of the data from the single sensors, we found that the reagents were distributed uniformly along the sensor region of the channel and remained so after drying.

### Simultaneous measurements with clinical samples

A cartridge with four microchannels was used. One microchannel was functionalized as a negative control. The remaining three channels were functionalized for LAA, glutamate, and choline assays. Fifteen microliters of clinically sourced human plasma samples were introduced into the cartridge with the preloaded reagents without any further dilution. Experiments were repeated twice. Microchannels were functionalized with dried reagents prior to each experiment. Experiments were performed immediately after completing cartridge functionalization.

### Signal processing

Signal processing can be divided into initial reaction rate determination followed by substrate concentration estimation.

To determine the initial reaction rate in a single microchannel, data were first visually inspected. Signals from sensing elements inside the same microfluidic channel were low pass filtered (normalized cutoff frequency: 0.1; 8th order) and spatially averaged (48 different sensors). Unresponsive sensors or sensors affected by strong artifacts were excluded from the averaging process. The resulting signal was then temporally averaged in 1-s nonoverlapping windows and fitted using a double exponential derived by the Michaelis–Menten model and Beer–Lambert law. The initial rate of the reaction was then calculated by differentiation of the measured signal.

The substrate concentration estimation was performed using the initial reaction rate and sample-specific parameters. For each sample, a negative control reaction was initiated between the sample, peroxidase, and color-changing reagents with no substrate-specific enzyme present to quantify nonspecific activity. The reaction rate obtained from the negative control (*r*_n_) was used as a background to adjust the reaction rate of the actual test (*r*_t_), as follows:

$$r_{\mathrm{t}}^ \ast = r_{\mathrm{t}} - r_{\mathrm{n}}$$

where *r*_t_* is the adjusted reaction rate of the test. Subtraction of the background can affect the performance of the assay, including the dynamic range, LOD and LOQ. However, each plasma sample used in this work had a different background since they came from different individuals. Cartridge to cartridge variations were also expected. The background correction takes into account these variations to yield comparable results.

The sensitivity was estimated using the two positive controls A and B, where known substrate concentrations [A] and [B], respectively, were added to the undiluted sample. The controls with concentrations [A] and [B] gave respective initial reaction rates *r*_a_ and *r*_b_ in the linear operating range of the platform. The rates *r*_a_ and *r*_b_ provided the sample-specific sensitivity (*S*′) of the apparatus according to the following formula:

$$S^{\prime} = \frac{{r_{\mathrm{b}} - r_{\mathrm{a}}}}{{\left[ {\mathrm{B}} \right] - [{\mathrm{A}}]}}\,{\mathrm{where}}\,\left[ {\mathrm{B}} \right] > [{\mathrm{A}}]\,\,{\mathrm{and}}\,\,r_{\mathrm{b}} > r_{\mathrm{a}}$$

By analogy, the sensitivity was also calculated using the following variants:

$$S^{\prime\prime} = \frac{{r_{\mathrm{b}} - r_{\mathrm{t}}}}{{\left[ {\mathrm{B}} \right] - [{\mathrm{T}}]}};\,S^{\prime\prime\prime} = \frac{{r_{\mathrm{a}} - r_{\mathrm{t}}}}{{\left[ {\mathrm{B}} \right] - [{\mathrm{T}}]}}$$

where *T* is the test sample with an unknown metabolite concentration [T]. Typically, we found that *S*′, *S*″, and *S*‴ had similar numerical values. Their average (*S*) was then used for substrate quantification. Note that it was not necessary to adjust *r*_a_ and *r*_b_ using *r*_n_ since *r*_n_ automatically cancels when computing the difference. [T] was estimated using linear regression to be:

$$[{\mathrm{T}}] = \frac{{r_{\mathrm{t}}^ \ast }}{S}$$

Additional details regarding signal processing can be found in the Supplementary Information.

## Supplementary information

Supplementary information - clean version
