# Supplementary material for: A monolithic single-chip point-of-care platform for metabolomic prostate cancer detection
Source: Microsyst Nanoeng. 2021 Mar 5;7:21. doi: 10.1038/s41378-021-00243-4 (PMC8433377; doi:10.1038/s41378-021-00243-4)
Supplement: Supplementary file 1 — Supplementary information - clean version [file 41378_2021_243_MOESM1_ESM.docx]

# **A monolithic single-chip point-of-care platform for metabolomic prostate cancer detection**

Supplementary Information

V.F. Annese^1^, S.B. Patil^1^, C. Hu^1^, C. Giagkoulovits^1^, M.A. Al-Rawhani^1^, J. Grant^1^, M. Macleod^2^, D.J. Clayton^3^, L.M. Heaney^4^, R. Daly^5^, C. Accarino^1^, Y.D. Shah^1^, B.C. Cheah^1^, J. Beeley^1^, T.R.J. Evans^6^, R. Jones^6^, M.P. Barrett^5,7^, D.R.S. Cumming^1^

*^1^ Electronics and Nanoscale Engineering, James Watt School of Engineering, University of Glasgow, Glasgow, G12 8QQ, UK*

*^2^ Beatson West of Scotland Cancer Centre, Glasgow, G12 0YN, UK*

*^3^ School of Science and Technology, Nottingham Trent University, Nottingham, NG11 8NF, UK*

*^4^ School of Sport, Exercise & Health Sciences, Loughborough University, Loughborough, LE11 3TU, UK*

*^5^ Glasgow Polyomics, College of Medical, Veterinary and Life Sciences, University of Glasgow, Glasgow, G61 1BD, UK*

*^6^ Institute of Cancer Sciences, University of Glasgow, Beatson West of Scotland Cancer Centre, Glasgow, G12 0YN, UK*

*^7^ Institute of Infection, Immunity and Inflammation, University of Glasgow, Glasgow, G12 8TA, UK*

## Cancer Metabolomics

There are metabolic differences between cancer and healthy cells thus metabolites that are modified by a cancer accumulate in human body fluids. Their altered levels have the potential to act as biomarkers to diagnose and monitor the disease [1]. Numerous studies of the levels of metabolomic biomarkers and their utility with respect to the diagnosis of different cancers have been published [2]–[4]. A review of cancer-related metabolites is presented in Table 1. Information from these studies was used in the design of the present work by selecting a small number of appropriate metabolites to measure on the POC platform.

Table 1. A review of cancer-related metabolites.

| **Cancer** | **Sample** | **Metabolites** |
| --- | --- | --- |
| **Breast** | Serum* | 2-hydroyglutarate [5], acetoacetate [6], beta-alanine [5], choline [7], fatty acid [8], glucose [9], glutamate [5], [6], glutamine [5], glycerol [6], glycerophosphocholine [7], histidine [6], [9], linoleic acid [8], lipids [9], mannose [6], n-acetyl glycoprotein [6], palmitic acid [8], phenylalanine [6], phosphocholine [7], pyruvate [6], steriatic acid [8], xanthine [5] |
|  | Tissue** | acetoacetate [10], histidine [10], glycerol [10], mannose [10], phenylalanine [10], pyruvate [10], linoleic acid [11], glutamate [10], glutamine [11], myoinositol [2], phosphoethanolamine [2], taurine [2], asparagine [12], [13], isoleucine [11], threonine [11] |
|  | Urine | 4-hydrolyphenylacetate [14], 5-hydrixyindoleacetic acid [14], homovanillate [14], urea [14] |
| **Colorectal** | Serum* | 2-Hydroxybutyrate [15], alanine [16], arginine [2], aspartic acid [15], [16], choline [17], cystamine [15], cysteine [2], fatty acid [2], glucose [2], glycin [17], glycine [16], histidine [16], inositol [17], isoleucine [16], kynurenine [15], lactate [17], leucine [17], lysine [16], methionine [16], oleamide [2], phenylalanine [17], phosphocholine [17], pyruvate [18], sarcosine [16], taurine [17], threonine [17], tryptophan [18], [19], tyrosine [16], [18], ultralong fatty acids [20], uridine [18], valine [16] |
|  | Tissue** | 2-aminobutyrate [21], 2-Hydroxybutyrate [21], 2-oxobutyrate [21], 5-Hydroxytryptamine [21], arginine [21], betaine [21], fatty acid [22], [23], glutamic acid [21], glutamine [22], [23], indoxyl [21], lactate [18], linoleic acid [21], N1-acetylspermidine [21], N-acetyl-5-hydroxytryptamine [21], nicotinic acid [21], proline [17], [21], symmetric dimethylarginine [21], threonine [21], uracil [21], urea [22]–[24], xanthine [21] |
| **Pancreatic** | Serum* | 3,6-dihydroxy-5-cholan-24-oic acid [3], 3-hydrolybuterate [2], 3-hydrolyisovalerate [2], 3-Hydroxybutyrate [25], [26], 3-hydroxyisovalerate [25], acetone [25], [27], alanine [28], arachidonic acid [29], arachidyl carnitine [30], butanoic acid [29], chenodeoxycholic acid [30], choline [28], citrate [27], creatine [25]–[27], cysteine [29], ethanol [25], formate [25], [27], glucose [25], [28], glutamate [25], glutamine [25], [29], glycerol [25], glycerol 2-phosphate [28], glycerol 3-phosphate [28], Glycholic acid [3], glycodeoxycholic acid [3], hydroxybutyrate [27], hypoxanthine [29], isoleucine [26], lactate [26], linoleic acid [28], lipids [27], lysine [29], malate [28], mannose [25], medium-chain acylcarnitines [31], myoinositol [28], N-acetyl glycoprotein [27], N-methylalanine [29], oleoyl carnitine [3], phenanthrenol [29], phenylalanine [25], [29], phosphatidylcholine [2], proline [25], quinaldic acid [30], sitosterol [30], tauro(ursodeoxy)cholic acid [29], tetradecanal oleamide [30], triglycerides [26], trimethylamine-N-Oxide [26], tyrosine [29] |
|  | Tissue** | Alanine [4], arachidonic acid [32], asparagine [4], choline [4], citrate [33], fatty acid [33], glutamic acid [4], glutamine [32], glycerophosphocholine [34], ketones [33], lactate [4], leucine [4], lysine [32], lysophosphatidylcholine [35], N-methylalanine [32], phenylalanine [32], phosphatidylcholine [35], phosphocholine [34], sphingolipid [33], sphingomyelin [35], taurine [4], tauro(ursodeoxy)cholic acid [32], valine [4] |
| **Ovarian, uterus, cervix** | Serum* | 2-Piperidinone [36], glycin [2], lysophosphatidylcholine [36], pyrimidine [2], tryoptophan [36] |
|  | Urine | 1-methylguanine [37], histidine [38], ketones [39], lactate [39], methylxanthine [37], mucin [38], N4-acetylcytidine [38], Nucleotide [38], proline [37], pseudouridine [38], pyridylacetic acid [37], succinic acid [38], theophylline [37], tryptophan [36], urate-3-ribonucleoside [38], uric acid [37], urocanic acid [37] |
| **Prostate** | Serum* | Alanine [40], [41], androsterone sulfate [42], arachidonoyl amine [42], arginine [40], [43], cholesterol [42], choline [44], citrate [40], [45], creatinine [46], [47], cysteine [42], dimethylheptanoyl carnitine [42], fatty acid [40], formate [42], glucose [43], glutamate [40], [42], glycine [42], isolithocholic acid [42], lactate [48], [49], leucine [42], lysine [42], [43], phenylalanine [43], [50] phosphocholine [42], proline [40], testosterone sulfate [42] |
|  | Tissue** | Alanine [50], arginine [51], asparagine [50], cholesterol [42], [51], [52], choline [42], [50], [53], citrate [42], cysteine [51], fatty acid [42], glutamate [42], glutamine [42], glycerol [42], [50], glycine [42], [53], lactate [42], [50], leucine [71], myoinositol [50], phenylalanine [42], phosphocholine [42], [50], proline [42], [50], pyrimidine [42], [50] |
|  | Urine | Alanine [50], choline, [50], citrate [50], creatinine [42], cysteine [42], fatty acid [42], glycerol [42], lactate [50], phosphocholine [50], pyrimidine [42] |
| **Oesophageal** | Serum* | adenosine monophosphate [54], NAD [54], acetoacetate [2], acetone [2], asparagine [51], aspartate [2], beta-hydrolybutyrate [55], citrate [55], creatine [2], cysteine [2], glucose [54], glutamate [2], glutamine [55], histidine [2], lactate [2], lactic acid [55], LDL [2], leucine [2], linoleic acid [55], lysine [55], methionine [55], myristic acid [55], phenylalanine [56], tryptophan [55], tyrosine [55], valine [55], VLDL [2], |
|  | Tissue** | Acylcarnitines [57], carnitine [57], fatty acid [57], lysophosphatidylcholine [57] |
| **Lung** | Serum* | 6-diaminopimelate [58], cholesteryl acetate [58], choline [59], fatty acid [59], [60], glutamine [61], lysophosphatidylcholine [58], [60], N-succinyl-2 [58], octanoylcarnitine [58], phosphatidylcholine [58], phosphatidylserine [58], sphingomyelin [60] |
|  | Tissue** | Choline [62], fatty acids [62], glutamine [63] |
| **Brain** | Tissue** | arachidonic acid [32], glycerophosphocholine [32], lactate [32], lysophosphatidylcholine [32], phosphatidylcholine [32] |
|  | CSF*** | 2-aminopimelic acid [64], citric acid [64], isocitric acid [64], methionine [64], serine [64], tyrosine [64], valine [64] |
| **Leukaemia** | Serum* | Acetone [65], alanine [65], arginine [65], cholesterol [65], [66], choline [65], creatine [65], cysteinyl-glycine [67], formate [65], glucose [65], glutamate [67], glycerol [66], histidine [65], lactate [65], [66], leucine [65], lysine [65], lysophosphatidylcholine [67], myoinositol [65], phenylalanine [65], phosphatidylcholine [67], phosphocholine [66], proline [65], pyruvate [66], trimethylamine-N-Oxide [65], tyrosine [65],  uric acid [66], uridine [66], valine [65] |
|  | Tissue** | Asparagine [12], [68], glutathione [69] |
| * This group includes blood, serum or plasma  ** This group includes tissue, cells or locally collected biological fluid (e.g. secretions)  *** Cerebrospinal fluid | | |

### Identifying a prostate cancer specific metabolic marker panel

Table 2 refines the range of potential metabolites shown in Table 1 to those known to be relevant to prostate cancer (PCa). Table 2 shows the link between PCa and metabolites using four sub-categories: associated risk or likelihood of recurrence; evidence of diagnostic capability; evidence of capability of discriminating between a malignant and a benign tumor; and demonstrated capability of providing information about cancer stage, including presence of eventual metastasis.

Since our objective was to devise a single test for multiple metabolites, the data from Table 2 was further refined as shown in Table 3 to consider only those metabolites that could be measured in blood. If metabolites that are found only in tissues or other fluids such as urine were used, a biopsy or multiple tests would be needed. Since the platform in this study relies on a colorimetric assay, we modelled the expected optical absorption characteristics of different candidate assays for metabolites using Matlab. The simulation took into account the Michaelis-Menten model for enzymatic substrate turnover using parameters from the BRENDA database [70], and the Beer-Lambert law for optical absorption. The simulated absorption for a given colorimetric reaction was paired to the characteristics of the photodiodes to assess the suitability of available assays for integration on to the platform [71].

It can be seen from Table 3 that no single blood metabolite that was likely to give a good signal was uniquely specific to PCa hence a more selective collaborative panel of metabolites was desirable.

Glutamate was selected because of its higher physiological concentration, potentially leading to a more reliable assay. Glutamine is metabolised in the glutaminolysis cycle to yield energy and glutamate [72]. During the tricarboxylic acid cycle, oxidative phosphorylation produces stored energy in the form of adenosine triphosphate. In cancer cells the cycle accelerates and as a consequence more glutamate is produced [72]–[74]. LAAs are intermediate products of the cycle and may therefore also be used as a biomarker for elevated glutamine metabolisation. The glutaminolysis cycle is known to increase fatty and nucleic acid synthesis that sustain cell proliferation in cancer.

Choline and sarcosine are essential compounds in fatty acids and cell membrane synthesis [75], thus increased levels of choline and sarcosine are thought to indicate biological transformation of cells linked to tumour cell proliferation. Choline was included in the panel as a distinctive measurement that is related to lipid transport and cell membrane synthesis [75]. As well as LAA, sarcosine concentration appears from Table 3 to be a highly specific metabolite for PCa among the relevant choices and therefore was included in the metabolic panel. Sarcosine has been found to be linked to colorectal cancer as well as PCa and the usefulness of sarcosine as a marker for PCa is debatable [74], but could be seen to be a viable candidate marker from the literature.

Owing to the dysregulation of glutamate, LAA, choline and sarcosine in tumour cell proliferation, and their suitability for working on the platform, these metabolites were selected for our panel.

Table 2. Summary of metabolites that have been linked to PCa in the literature. Metabolite correlation with cancer has been divided into the type of sample (serum*, tissue**, urine) and cancer stage.

| **Metabolite** | | **Sample** | **Risk or recurrence** | **Diagnosis** | **Malignancy (vs benignancy)** | **Staging** |
| --- | --- | --- | --- | --- | --- | --- |
| Androsterone sulfate | | Serum* |  | ↑ [42] |  |  |
|  |  | Tissue** |  |  |  |  |
|  |  | Urine |  |  |  |  |
| Arachidonoyl amine | | Serum* |  | ↑ [42] |  |  |
|  |  | Tissue** |  |  |  |  |
|  |  | Urine |  |  |  |  |
| Cholesterol | | Serum* |  |  |  | ↑ [42] |
|  |  | Tissue** |  |  |  | ↑[42] |
|  |  | Urine |  |  |  |  |
| Choline | | Serum* | ↑ [44] | ↑ [44] |  |  |
|  |  | Tissue** |  | ↑ [42], [50] | ↑ [42], [50] | ↑ [42], [51], [53] |
|  |  | Urine |  | ↑ [50] | ↑ [50] |  |
| Citrate | | Serum* | ↔ [40] |  | ↔ [40] | ↓ [40], [76] |
|  |  | Tissue** | ↓ [42] | ↓ [42] | ↓ [42] |  |
|  |  | Urine |  | ↓ [50] |  | ↓ [50] |
| Creatinine | | Serum* | ↑ [46] |  |  | ↑ [47] |
|  |  | Tissue** |  |  |  |  |
|  |  | Urine |  |  | ↓ [42] |  |
| dimethylheptanoyl carnitine | | Serum* |  | ↓ [42] |  |  |
|  |  | Tissue** |  |  |  |  |
|  |  | Urine |  |  |  |  |
| Fatty acid | | Serum* | ↔ [40] |  | ↔ [40], [42] | ↔ [40] |
|  |  | Tissue** |  |  |  | ↔[42] |
|  |  | Urine |  |  | ↔ [42] |  |
| Formate | | Serum* |  |  | ↔ [42] | ↔ [42] |
|  |  | Tissue** |  |  |  |  |
|  |  | Urine |  |  |  |  |
| Glucose | | Serum* |  |  | ↑ [43] |  |
|  |  | Tissue** |  |  |  |  |
|  |  | Urine |  |  |  |  |
| Glycerol | | Serum* |  |  |  |  |
|  |  | Tissue** |  | ↑[42], [50] | ↔ [42] | ↑ [50] |
|  |  | Urine |  | ↑ [42], [50] |  | ↑ [50] |
| Isolithocholic acid | | Serum* |  | ↓ [42] |  |  |
|  |  | Tissue** |  |  |  |  |
|  |  | Urine |  |  |  |  |
| **L-amino acids (LAA)** | Alanine | Serum* |  | **↔** [41] |  | ↓ [40] |
|  |  | Tissue** |  |  | ↑ [50] |  |
|  |  | Urine |  |  | ↑ [50] |  |
|  | Arginine | Serum* |  |  | ↑ [43] | ↑ [40] |
|  |  | Tissue** |  |  |  | ↑ [51] |
|  |  | Urine |  |  |  |  |
|  | Asparagine | Serum* |  |  |  |  |
|  |  | Tissue** |  |  | ↑ [51] | ↑ [50] |
|  |  | Urine |  |  |  |  |
|  | Cysteine | Serum* | ↔ [42] |  |  |  |
|  |  | Tissue** |  |  | ↑ [51] |  |
|  |  | Urine | ↔ [42] |  |  |  |
|  | Glutamate | Serum* | ↔ [40] | ↑[42], [77] | ↑ [40], [43], [77] | ↑ [40], [42], [50], [77] |
|  |  | Tissue** |  | ↔ [42] | ↑ [42] | ↑ [50] |
|  |  | Urine |  |  |  |  |
|  | Glutamine | Serum* |  |  |  |  |
|  |  | Tissue** | ↔[42] | ↔[42] |  |  |
|  |  | Urine |  |  |  |  |
|  | Glycine | Serum* |  | ↑ [42] |  |  |
|  |  | Tissue** |  |  | ↑ [42] | ↑ [53] |
|  |  | Urine |  |  |  |  |
|  | Leucine | Serum* |  |  |  | ↑ [42] |
|  |  | Tissue** |  |  | ↔ [42] | ↑ [50] |
|  |  | Urine |  |  |  |  |
|  | Lysine | Serum* |  | ↔ [42] | ↑ [43] | ↓ [42] |
|  |  | Tissue** |  |  |  |  |
|  |  | Urine |  |  |  |  |
|  | Phenylalanine | Serum* |  |  | ↑ [43] | ↑ [50] |
|  |  | Tissue** |  |  |  | ↑ [42] |
|  |  | Urine |  |  |  |  |
|  | Proline | Serum* |  |  | ↑ [40] | ↑ [40] |
|  |  | Tissue** |  | ↑ [42] |  | ↑ [50] |
|  |  | Urine |  |  |  |  |
|  | Serine | Serum* |  |  |  |  |
|  |  | Tissue** |  |  |  | ↓ [51] |
|  |  | Urine |  |  |  |  |
|  | Tryptophan | Serum* | ↔ [40] |  | ↔ [40] | ↑ [40] |
|  |  | Tissue** |  |  |  |  |
|  |  | Urine |  |  |  |  |
|  | Tyrosine | Serum* |  |  |  | ↔ [42] |
|  |  | Tissue** |  |  |  |  |
|  |  | Urine |  |  |  |  |
|  | Valine | Serum* |  |  | ↑ [43] | ↑ [42] |
|  |  | Tissue** |  | ↓ [50] |  |  |
|  |  | Urine |  |  |  |  |
| Lactate | | Serum* |  | ↑ [48], [49] |  |  |
|  |  | Tissue** |  | ↑ [42], [50] | ↑ [50] |  |
|  |  | Urine |  | ↑ [50] | ↑ [50] |  |
| Myoinositol | | Serum* |  |  |  |  |
|  |  | Tissue** |  |  | ↑ [51] | ↑ [42], [50] |
|  |  | Urine |  |  |  |  |
| Phosphocholine | | Serum* |  | ↔ [42] |  |  |
|  |  | Tissue** |  | ↑ [42], [50] | ↔ [42] | ↑ [50] |
|  |  | Urine |  | ↑ [50] |  |  |
| Pyrimidine | | Serum* |  |  |  |  |
|  |  | Tissue** |  | ↓ [50] |  |  |
|  |  | Urine |  | ↓ [42], [50] |  |  |
| Sarcosine | | Serum* |  | ↑ [41] |  |  |
|  |  | Tissue** |  | ↑ [42] | ↑ [42], [50], [51] | ↑ [42], [50], [53], [78] |
|  |  | Urine |  |  | ↑ [42], [50], [51] | ↑ [50], [51], [53], [78] |
| Spermine | | Serum* |  |  |  | ↓ [8], [42], [51] |
|  |  | Tissue** | ↓ [42] | ↓ [50] | ↓ [50] | ↓ [8], [42], [51] |
|  |  | Urine |  | ↓ [50] | ↓ [50] |  |
| Taurine | | Serum* |  |  |  | ↑ [50] |
|  |  | Tissue** |  |  | ↔ [42] | ↑ [42] |
|  |  | Urine |  |  |  |  |
| Urea | | Serum* | ↔ [40] |  | ↔ [40] | ↑ [40], [45], [47] |
|  |  | Tissue** |  |  |  |  |
|  |  | Urine |  |  |  |  |
| xanthine | | Serum* |  |  | ↔ [40] | ↑[40], [51] |
|  |  | Tissue** |  |  |  |  |
|  |  | Urine |  |  |  |  |
| Testosterone sulfate | | Serum* |  | ↓ [42] |  |  |
|  |  | Tissue** |  |  |  |  |
|  |  | Urine |  |  |  |  |
| ↑: increased concentration level  ↓: decreased concentration level  ↔: altered concentration level but the trend is not specified or not easily reportable  * This group includes blood, serum, or plasma  ** This group includes tissue, cells or locally collected biological fluid (e.g. secretions) | | | | | | |

Table 3 Determination of the proposed PCa-specific blood metabolites panel.

| **PCa-related blood metabolites** | | **On-chip detection feasibility ^1^** | **Metabolite has also been linked to:** | | | | | | | | | **Specificity to PCa^4^** |
| --- | --- | --- | --- | --- | --- | --- | --- | --- | --- | --- | --- | --- |
|  |  |  | **Breast Cancer** | **Colorectal Cancer** | **Pancreas Cancer** | **Ovarian,  Uterus Cervix cancer** | | **Oesophageal Cancer** | **Lung Cancer** | **Leukaemia** | **Brain Cancer** |  |
| Choline | | High | ✓ | ✓ | ✓ |  | |  | ✓ | ✓ |  | Low |
| Formate | | High |  |  | ✓ |  | |  |  | ✓ |  | Medium |
| Glucose | | High | ✓ | ✓ | ✓ |  | | ✓ |  | ✓ |  | Low |
| Lactate | | High |  | ✓ | ✓ |  | | ✓ |  | ✓ | ✓ | Low |
| Sarcosine | | High |  | ✓ |  |  | |  |  |  |  | High |
| Total LAA^2^ | | High |  | ✓ |  |  | | ✓ |  |  |  | Medium |
| **L-amino acids (LAA)** | Alanine | High | ✓ | ✓ | ✓ |  | |  |  | ✓ |  | Medium |
|  | Glutamate | High | ✓ |  | ✓ |  | | ✓ |  | ✓ |  | Medium |
|  | Glutamine^3^ | High | ✓ |  | ✓ |  | | ✓ | ✓ |  |  | n.d.^3^ |
|  | Leucine | High |  | ✓ |  |  | | ✓ |  | ✓ |  | Medium |
|  | Lysine | High |  | ✓ | ✓ |  | | ✓ |  | ✓ |  | Medium |
|  | Phenylalanine | High | ✓ | ✓ | ✓ |  | | ✓ |  | ✓ |  | Low |
|  | Tryptophan | High |  | ✓ |  | ✓ | | ✓ |  |  |  | Medium |
|  | Tyrosine | High |  | ✓ | ✓ |  | | ✓ |  | ✓ |  | Medium |
|  | Arginine | Medium |  | ✓ |  |  | |  |  | ✓ |  | Medium |
|  | Asparagine^3^ | Medium |  |  |  |  | | ✓ |  |  |  | n.d.^3^ |
|  | Aspartic acid^3^ | Medium |  | ✓ |  |  | | ✓ |  |  |  | n.d.^3^ |
|  | Cysteine | Medium |  | ✓ | ✓ |  | | ✓ |  |  |  | Medium |
|  | Glycine | Medium |  | ✓ |  |  | |  |  |  |  | High |
|  | Histidine^3^ | Medium | ✓ | ✓ |  |  | |  |  |  |  | Medium |
|  | Isoleucine^3^ | Medium |  | ✓ | ✓ |  | |  |  |  |  | Medium |
|  | Methionine^3^ | Medium |  | ✓ |  |  | | ✓ |  |  |  | Medium |
|  | Proline | Medium |  |  | ✓ |  | |  |  | ✓ |  | Medium |
|  | Serine^3^ | Medium |  |  |  |  | |  |  |  |  | n.d.^3^ |
|  | Threonine^3^ | Medium |  | ✓ |  |  | |  |  |  |  | n.d.^3^ |
|  | Valine | Medium |  | ✓ |  |  | | ✓ |  | ✓ |  | Medium |
| Cholesterol | | Medium |  |  |  |  | |  |  | ✓ |  | High |
| Citrate | | Medium |  |  | ✓ |  | | ✓ |  |  |  | Medium |
| Creatinine | | Medium |  |  |  |  | |  |  |  |  | High |
| Fatty acid | | Medium | ✓ | ✓ |  |  | |  | ✓ |  |  | Medium |
| Phosphocholine | | Medium | ✓ | ✓ |  |  | |  |  | ✓ |  | Medium |
| Spermine | | Medium |  |  |  |  | |  |  |  |  | High |
| Urea | | Medium |  |  |  |  | |  |  |  |  | High |
| xanthine | | Medium | ✓ |  |  |  | |  |  |  |  | High |
| Androsterone sulphate | | Low |  |  |  |  | |  |  |  |  | High |
| Arachidonoyl amine | | Low |  |  |  |  | |  |  |  |  | High |
| Dimethyl heptanoyl carnitine | | Low |  |  |  |  | |  |  |  |  | High |
| Isolithocholic acid | | Low |  |  |  |  | |  |  |  |  | High |
| Taurine | | Low |  | ✓ |  |  | |  |  |  |  | High |
| PCa-specific metabolites panel | | | | | | | | | | | | |
| Sarcosine + LAA | | High |  | ✓ |  |  |  | |  |  |  | High |
| Sarcosine + LAA + Glutamate | | High |  |  |  |  |  | |  |  |  | High |
| LAA + Glutamate + Choline | | High |  |  |  |  |  | |  |  |  | High |
| LAA + Glutamate + Choline + Sarcosine | | High |  |  |  |  |  | |  |  |  | High |
| ^1^ **High**: a) The metabolite is suitable for the colorimetric determination. b) All required reagents for the colorimetric assays are commercially available; c) Physiological concentration is expected to produce a detectable signal.  **Medium**: One of the above criteria is not met. **Low**: two or more of the above criteria are not met.  ^2^ LAA profile was considered linked to cancer types where 10 or more amino acids showed correlation.  ^3^ No evidence was found linking this metabolite to PCa.  ^4^ Calculated as the number of additional cancer types linked to the specific metabolite. **High**: ≤1, **Medium**: 2-4; **Low**: ≥5 | | | | | | | | | | | | |

## Platform Development

## The Cartridge

The cartridge integrated biochemical reagents, the CMOS chip and the passive microfluidics. All the parts were built into a ceramic chip package.

All the selected metabolites were detected with a two-stage colorimetric approach. Substrate specific enzymes were used to catalyse a reaction with the target analyte to produce hydrogen peroxide. The enzymatic reactions we used were [70]:

$$\boldsymbol{LAA:}L-amino acid+H_{2}O+O_{2} \underset{\to}{L-amino acid oxidase (LAAOx)}\alpha-oxo acid+ {NH}_{3}+ H_{2}O_{2}$$

$$\boldsymbol{Glutamate:}Glutamate +H_{2}O+O_{2} \underset{\to}{Glutamate Oxidase (GLOx)}2-oxoglutarate+ {NH}_{3}+ H_{2}O_{2}$$

$$\boldsymbol{Choline:}Choline +H_{2}O+O_{2} \underset{\to}{Choline oxidase (COx)}betaine+ H_{2}O_{2}$$

$$\boldsymbol{Sarcosine:}Sarcosine +H_{2}O+O_{2} \underset{\to}{Sarcosine oxidase (SOx)}glycine+ formaldehyde+H_{2}O_{2}$$

The H_2_O_2_ that is produced in each case is proportional to the concentration of the analyte of interest. The H_2_O_2_ was then monitored by a colorimetric probe that changed its absorbance characteristic according to the H_2_O_2_ concentration. The reaction used to probe for H_2_O_2_ was:

$$\boldsymbol{Quinone-imine}: Phenol+4-Aminoantipyrine +{2H}_{2}O_{2}\underset{\to}{Peroxidase \left( HRP \right)}Quinone-imine+{2H}_{2}O$$

For each metabolite test, two enzymatic reactions were therefore required. However, the ratio of the concentration of the enzymes within the same reaction chain was tuned so that the absorbance properties of the two-stage assay was proportional to the concentration of the metabolite in question.

The change in the light transmittance of the sample was measured using a custom CMOS chip [71]. The CMOS chip, shown in Figure 1(a), is a 16 x 16 array of sensor elements. Each sensor element integrates a photodiode, an ion sensitive field effect transistor and a single photon avalanche diode. For this work only the photodiodes were used. Interface electronics can address the photodiodes individually or simultaneously. Each element has dimensions of 100×100 μm^2^ thus the whole array occupies a total area of approximately 1.6 x 1.6 mm in the centre of the chip. The entire chip has an area of 3.4 x 3.6 mm (see Figure 1(b)). 64 contact pads are equally distributed in columns of 32 on the left and right side of the chip. Alignment marks (crosses and squares) are symmetrically placed on the top and bottom edges of the sensor array. The CMOS process selected for the fabrication of the chip was a 0.35 μm high voltage process with four metal layers (‘H35B4’) from austriamicrosystems. After fabrication, bare chips were diced so that they could be wire-bonded into a ceramic pin grid array with 120 pins to form the base of the cartridge.

The integration of passive microfluidics with the CMOS chip was achieved using soft lithography and injection moulding. The design of the passive microfluidics network was achieved through simulation and a trial and error experimental approach. Simulations were performed using a custom Matlab model for the optimisation of the: filling time; capillary flow rate; fluidic resistance of the microfluidic channel for a given sample type (water, human serum, plasma or blood); channel geometry; and material wetting properties. The process used for the integration of the microfluidics is described in the material and methods section of the paper. The fabrication steps used to make the microfluidics-on-chip structures are shown in more detail in Figure 2. Four parallel microfluidic channels were integrated on to each chip.

| 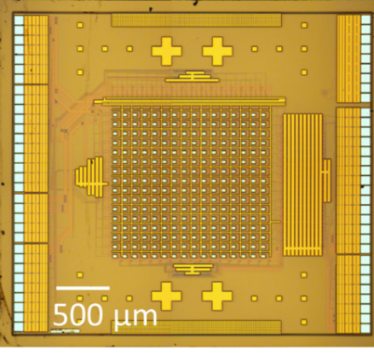 | 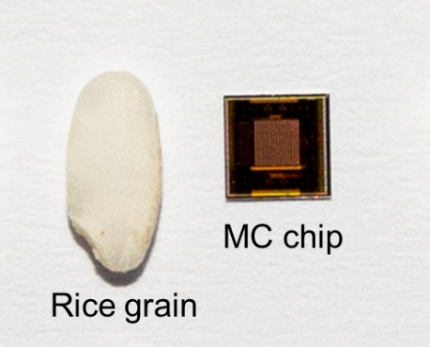 |
| --- | --- |
| (a) | (b) |
| Figure 1. (a) A micrograph of the CMOS chip showing its layout and (b) the CMOS chip, that is 3.4 x 3.6 mm, compared to a grain of rice grain. | |


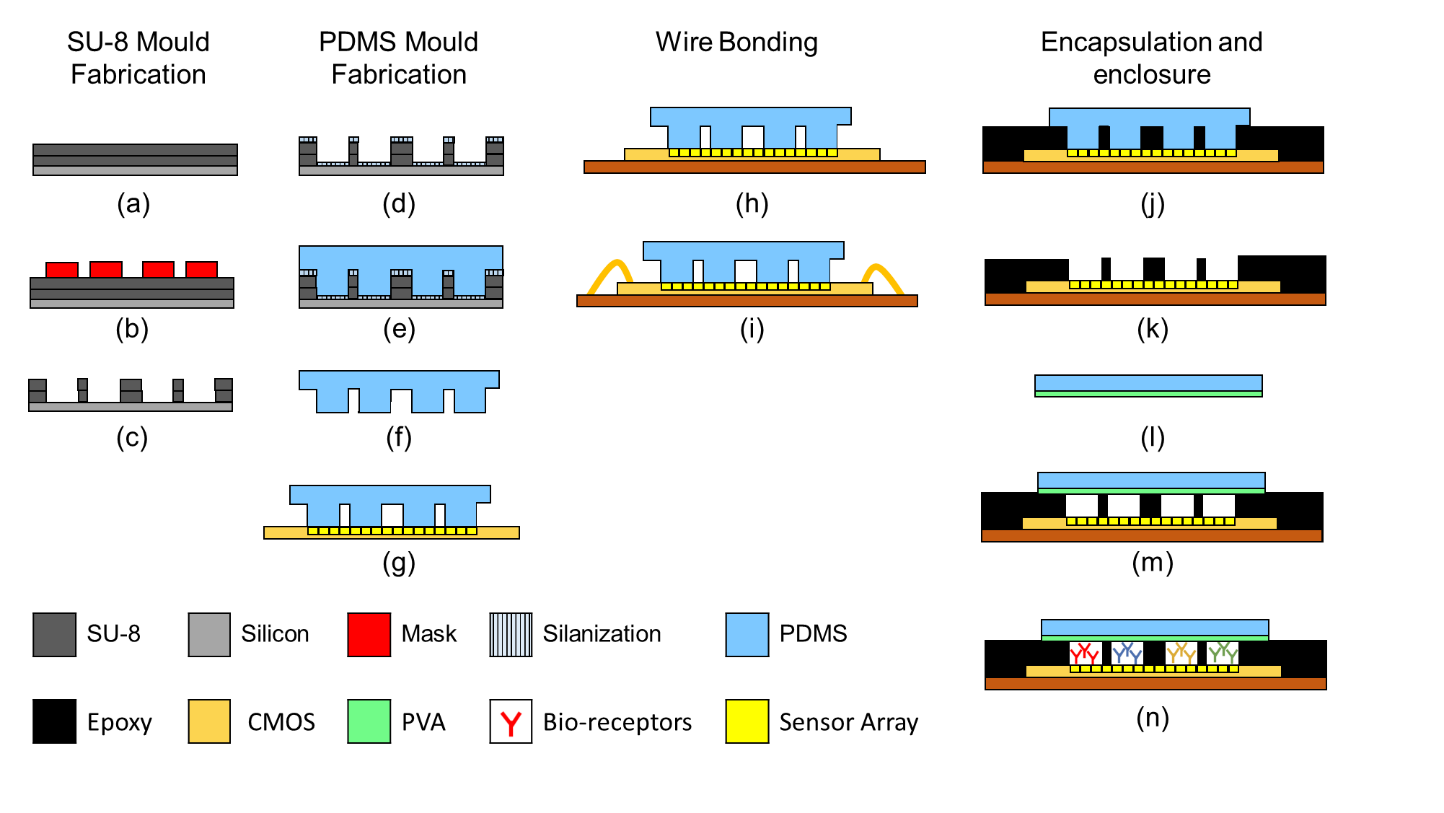


Figure 2. (a) A double layer of SU-8 3050 is spin-coated onto a silicon wafer. (b) Photolithography process. (c) Development and final SU-8 microstructure. (d) The surface of the mould is silanized. (e) PDMS is cast onto the mould. (f) PDMS is cured and peeled off the mould. (g) PDMS microstructure is temporarily bond onto the chip. (h) The chip is permanently bonded to the chip package. (i) The chip is wire-bonded. (j) Epoxy channels are fabricated using injection moulding. Epoxy also encapsulates the wire bonds. (k) The PDMS microstructure is removed. (l) A planar slab of PDMS is PVA coated. (m) The PVA-coated PDMS slab is bonded onto the epoxy microstructure. (n) Enzymatic reagents are loaded into the microchannels.

## The Reader

The reader is composed of a custom-PCB, a ST Nucleo F334R8 microcontroller board and a data communication module. The cartridge is fitted to the PCB using a zero-insertion force (ZIF) socket. The PCB provided a 3.3 V power supply to the chip and the interface to the microcontroller board. The microcontroller digitally addressed the array elements, digitised the data and transmitted it to the GUI via the communications module. The ST Nucleo F334R8 board integrates a 32-bit ARM Cortex CPU, 64kb flash memory, 12kb SRAM and a 12-bit ADC that was programmed with custom firmware [79]. The reader was connected to the GUI device via a USB link for power and ground. To increase the communication speed between the reader and the GUI a FT231X Universal Asynchronous Receiver/Transmitter (UART) with a Baud rate of 921,600 (Sparkfun electronics) was plugged into the PCB. The custom firmware for the microcontroller was developed on the ‘mbed’ online compiler using C++ [80]. The firmware implemented a continuous loop performing sensor reset, sequential reading of the photodiodes in the array, digitisation and transmission of the data. The PCB, UART module and the microcontroller board, that were stacked together to complete the reader, are individually shown in Figure 3.

## The Graphical User Interface (GUI)

The GUI was developed using the Matlab-based graphic user interface development tool (GUIDE) that can run on any Microsoft-based portable device. In this work, a laptop (HP EliteBook 830 G5) was used to host the GUI. The GUI receives binary data from the reader, and it is used for data collection, visualisation, processing, and analysis. A flow chart of the primary operations performed by the GUI is shown in Figure 4(a). The GUI has two modes of operation: data-acquisition and data-analysis. When working in the data acquisition mode, the GUI is used in conjunction with the reader and the cartridge to collect, display and save data. This is the modality used for monitoring colorimetric reactions. The data analysis mode was used to process data once an experiment was completed.

| 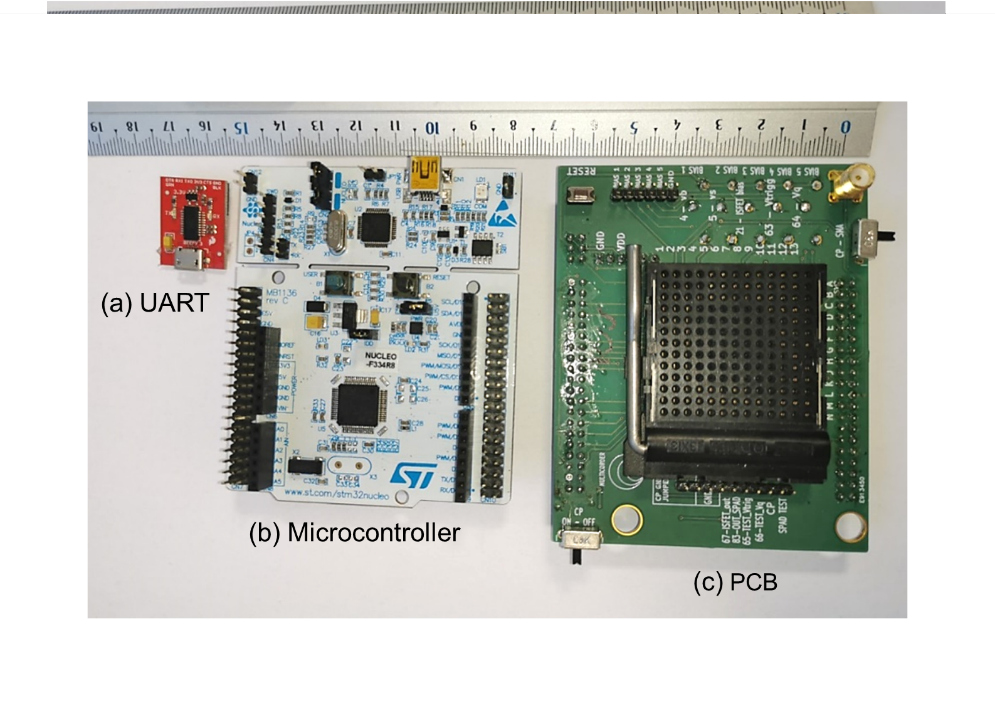 |
| --- |

Figure 3. (a) UART module, (b) microcontroller board and (c) PCB with ZIF socket.

| 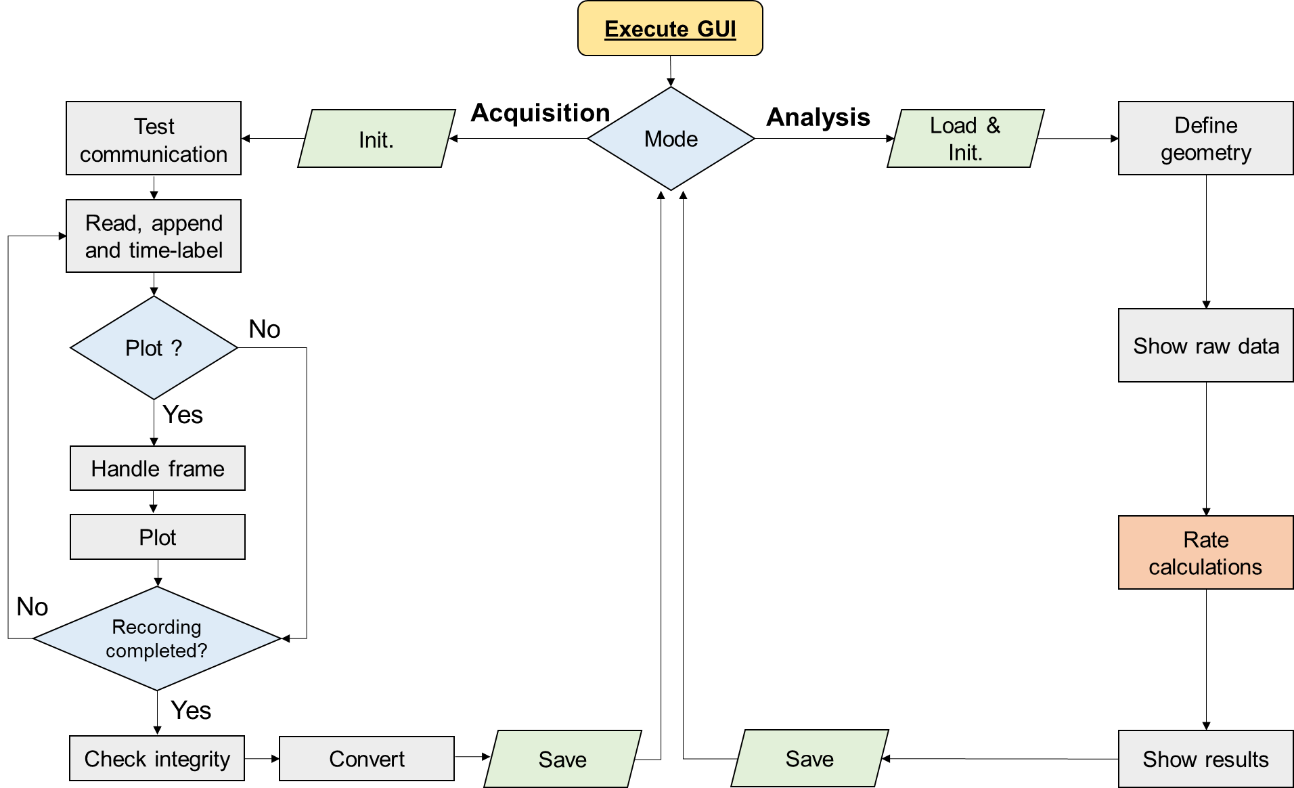 | |
| --- | --- |
| (a) | |
|  | |
| 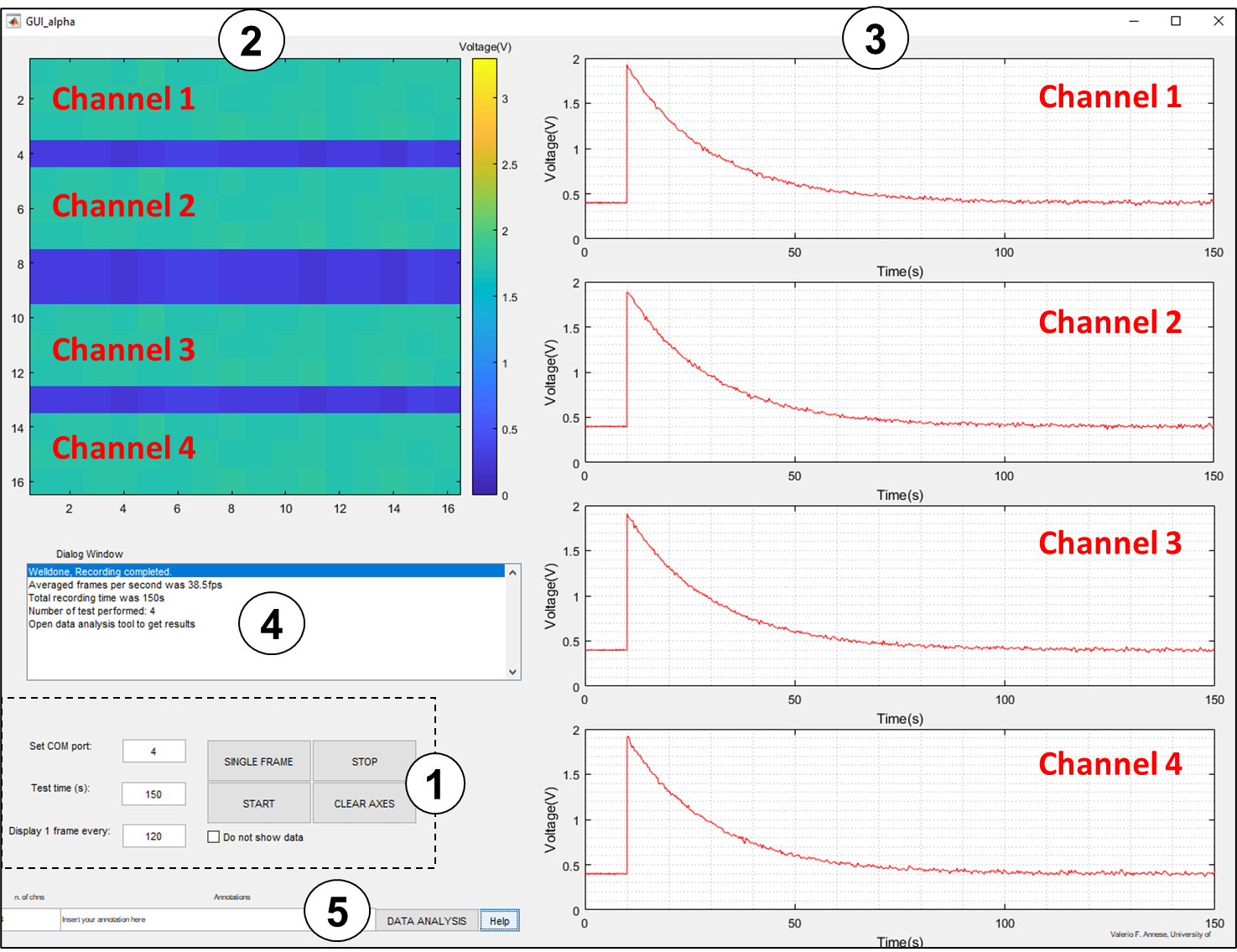 | 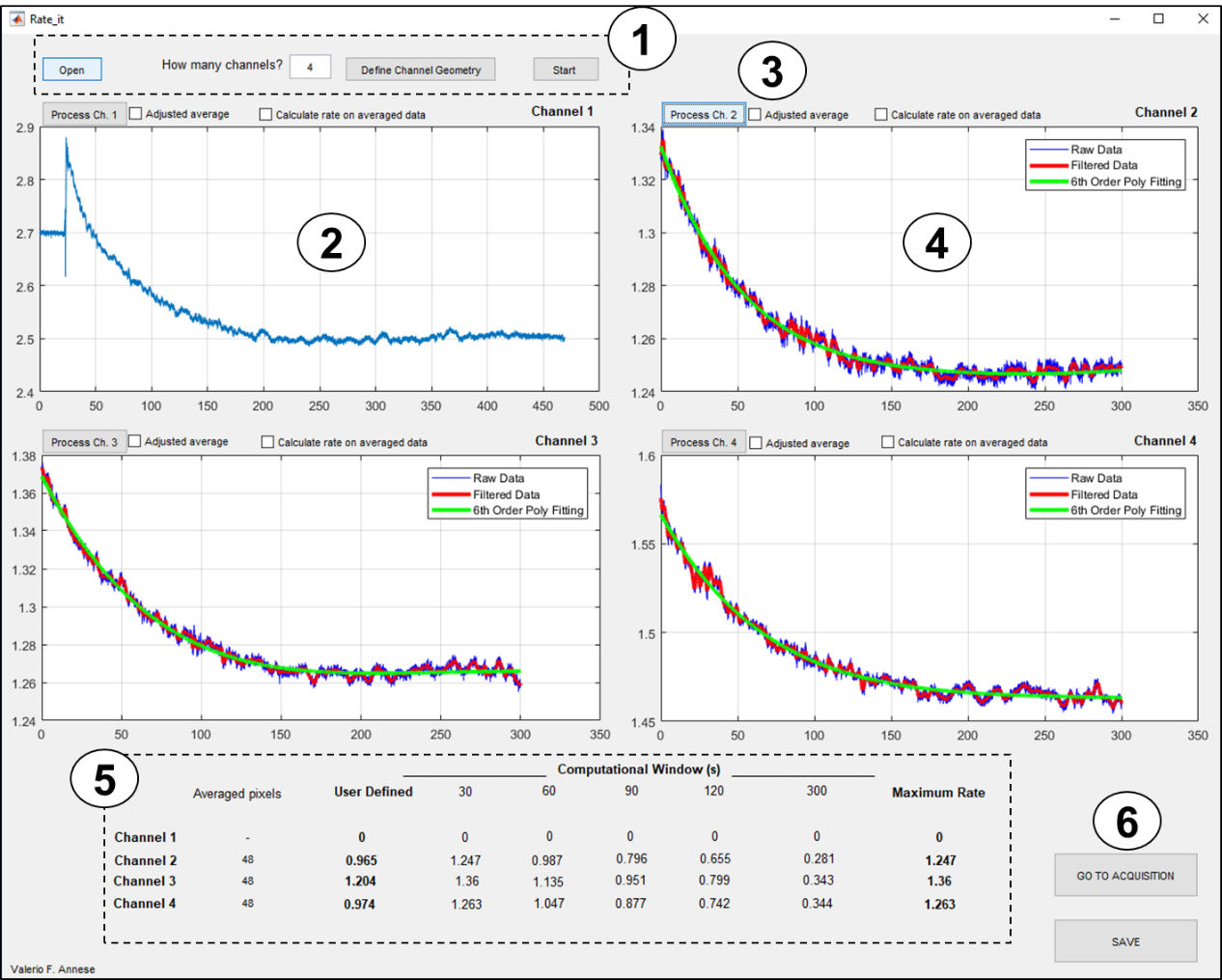 |
| (b) | (c) |

Figure 4. (a) Flow chart of the GUI operation. The GUI can operate in acquisition (left) or analysis (right) mode. In the acquisition mode the GUI is interfaced with the reader by USB link for real-time visualisation, handling and storage of the sensor outputs. In the analysis mode the GUI is used to process and visualise data offline. (b) GUI data acquisition mode. 1) Control panel; 2) Frame visualisation; 3) Single-pixel representation; 4) Dialog window; 5) Go to data analysis. (c) GUI in data analysis mode. 1) Control panel; 2) Raw data visualisation; 3) Start processing button for single-channel; 4) Processed data visualisation; 5) Rates (mVs^‑1^); 6) Save and go to data-acquisition mode.

When used in data acquisition mode, the GUI executed the operations on the left-hand branch of the flow chart shown in Figure 4(a). The user was able to modify the test duration, start/stop the recording, visualise real-time data and save the raw data using the custom control panel shown in Figure 4(b). Each scan of the array, referred to as a frame, was stored with a time label to create a vector. The recorded time was used during signal processing to determine the reaction rate. Data frames and their respective time labels with unexpected size (for example due to a transmission error) were not used.

When used in data analysis mode, the GUI executed the operation on the right-hand branch of the flow chart shown in Figure 4(a). Data from each microchannel on the chip was processed individually using the custom control panel shown in Figure 4(b). Data analysis was composed of three sub-routines: data preparation, noise reduction and rate calculation. Figure 5 shows the flow-chart of the numerical operations leading to the calculation of the initial reaction rate.

- **Data preparation**. The location of the sensors in the channel to be processed was defined. The starting time of the reaction was then identified. Usually, this was visible from raw data owing to the sudden optical transmittance variation induced by the sample introduction into the cartridge. Data was then recorded for a period of up to 300 s. Data from unresponsive photodiodes, presenting obvious artefacts (e.g. an air bubble), were excluded.
- **Noise reduction.** The noise sources in the CMOS sensor array can be divided into random stochastic noise and fixed pattern noise (FPN) [81]. Random noise is a combination of noise components (thermal, shot and flicker), noise in addressing circuit and noise in the ADC. FPN is the variation of the signal from sensor-sensor in an array and is invariant when the same stimulus is applied. Raw data was filtered using an 8^th^ order low pass filter (normalised cut‑off frequency: 0.1). Precautions were taken to avoid any signal distortion at the start and finish of each data stream. Data from sensors in the same microchannel was averaged to reduce the standard deviation as a consequence of FPN. A rolling average over a one second non-overlapping window of the data was then calculated. Assuming that both the noise from a single sensor and the variation of the characteristics over an entire array had a Gaussian distribution, the averaging process reduced the standard deviation of a factor √N, where N was the number of population in the average. Typically, N = 1728 [82]. The time vector was similarly averaged over a one second non-overlapping window of the data. The averaging process led to the generation of one averaged sensor output and a time vector per microchannel. The averaged sensor output as a function of time was then fitted into a double exponential model using fixed time-windows.
- **Rate calculation.** Reactions rates were calculated by differentiation of the signal with respect to time. The initial reaction rate was thus determined. The decomposition of the signal in different time windows allowed the automatic calculation of the rate. It was found from experiments that that it was always possible to determine the initial reaction rate within 2 minutes hence we concluded that a test duration of 2 minutes was sufficient.


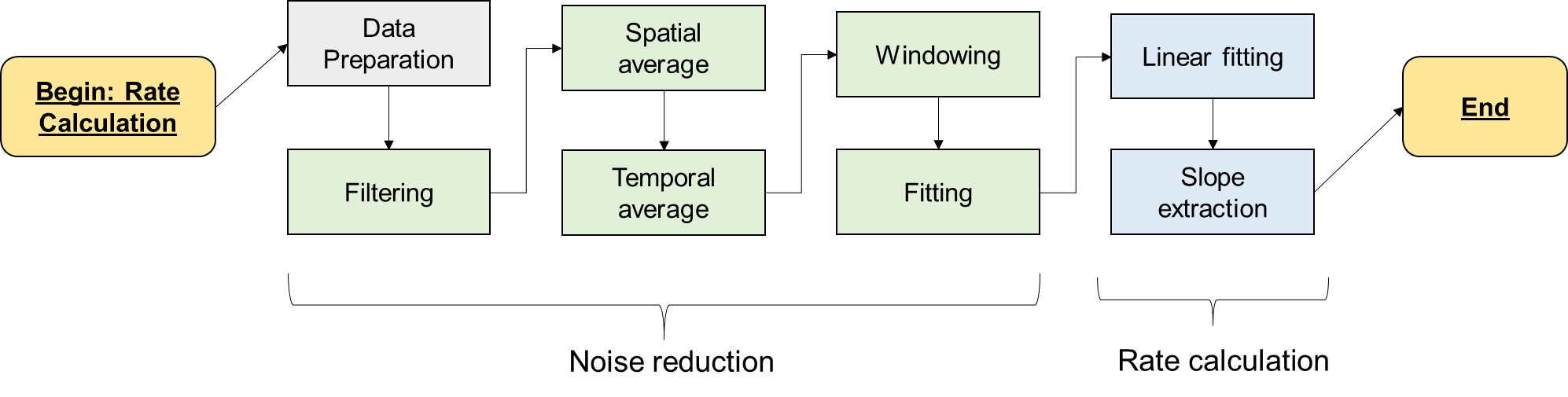


Figure 5. Flow chart diagram of the operations performed for the initial reaction rate calculation.

## Supplementary Information on Clinical Trials

Experiments were performed under ethical approval (reference number: 10/S0704/18 issued by the West of Scotland Research Ethics Service). Materials and methods related to the clinical trials have been described in the paper. Here we report the full dataset of the measurements (Table 5) and correlation graphs showing how the different metabolites cross-correlated (Figure 6).

To confirm that the platform was working correctly LAA, glutamate, choline, and sarcosine concentrations in samples from the healthy subject group were independently analysed at Loughborough University. Plasma samples were shipped under a specific material transfer agreement between the University of Glasgow and Loughborough University. All the metabolites were tested using commercially available colorimetric or fluorometric plate-based assays following manufacturer instructions (product codes: ab65347, ab138883, ab219944, ab65338, Abcam, Cambridge, UK). Choline was also analysed by ultra-performance liquid chromatography-tandem mass spectrometry (MS/MS) using an Acquity liquid chromatograph coupled to a Quattro Ultima triple quadrupole mass spectrometer (Waters, Wilmslow, UK).

Figure 7 shows a comparison of metabolite concentrations measured with the platform used in this work and with the above-mentioned methods. For each sample, metabolite and testing method, concentrations were normalised to the average levels in the group. All four metabolites had comparable results with all the above-mentioned methods. Choline readings using the platform (see Figure 7(e) and (f)) showed higher correlation with MS/MS measurements (R=0.8) than with data obtained with the commercial fluorescence kit (R=0.5). It is well-known that MS/MS provides more accurate results than the commercial assay kits. The data therefore suggests that measurements with the platform are more accurate than the commercial kit but have lower precision (larger standard deviation). MS/MS analysis for all the metabolites was not possible with the resources available.

An exact match was not expected. Factors that might have affected the comparison studies were:

- Samples tested in the third-party laboratory had to be transported a long-distance that might have affected the concentration of the metabolites.
- The substrate concentration was estimated using different techniques. The platform used sample-specific parameters, as discussed in the methods section of the paper. The reference methods used a ‘one-fit-all’ calibration curve obtained with calibrated solutions.
- The platform and the reference methods used different working principles. It has been shown that measurements of serum metabolites made using different methods might have discrepancies because of chemical interference, especially in the presence of anticoagulants, such as EDTA [83].

Table 4. Summary of the target metabolites for the selected applications

| **Analyte** | **Physiological range (µM)** | **Application** | **Detection method** | **Enzyme** | **Km** [70] |
| --- | --- | --- | --- | --- | --- |
| LAAs profile | 1700 - 4600 | ↑ in PCa | Colorimetric | LAAOx | 0.17 – 116.5 |
| Glutamate | 40-150 | ↑ in PCa | Colorimetric | GlOx | 0.15 - 10 |
| Choline | 10-40 | ↑ in PCa | Colorimetric | COx | 0.05 – 213 |
| Sarcosine | 0-20 | ↑ in PCa | Colorimetric | SOx | 0.01 – 142.3 |

Table 5. Clinical Trials results on control and cancer groups.

| **Group** | **Sample ID** | | **LAA** | | **Glutamate** | | | **Choline** | | | **Sarcosine** | | |
| --- | --- | --- | --- | --- | --- | --- | --- | --- | --- | --- | --- | --- | --- |
|  |  |  | Avg. µM | Std. % | Avg. µM | | Std. % | Avg. µM | Std. % | | Avg. µM | Std. % | |
| non-PCa | 1 | | 1961 | 16.2 | 42.5 | | 16.9 | 15.4 | 11.5 | | 15.1 | 23.5 | |
|  | 2 | | 2493 | 9.5 | 67.1 | | 44.7 | 8.7 | 34.0 | | 7.5 | 9.8 | |
|  | 3 | | 1972 | 29.4 | 30.7 | | 28.5 | 2.3 ^(<LOQ)^ | 78.7 | | 7.0 | 21.8 | |
|  | 4 | | 3167 | 10.4 | 34.2 | | 8.8 | 10.2 | 39.9 | | 18.8 | 38.1 | |
|  | 5 | | 1983 | 20.8 | 39.5 | | 14.5 | 14.8 | 54.5 | | 7.4 | 32.2 | |
|  | 6 | | 2187 | 5.4 | 47.1 | | 32.0 | 11.3 | 36.0 | | 5.1 | 44.6 | |
|  | 7 | | 1780 | 15.5 | 37.6 | | 53.0 | 3.2 ^(<LOQ)^ | 32.0 | | 12.4 | 63.5 | |
|  | 8 | | 1213 | 13.1 | 21.9 | | 4.1 | 9.4 | 17.5 | | 12.2 | 45.6 | |
|  | 9 | | 1390 | 14.4 | 40.8 | | 8.7 | 8.4 | 86.9 | | 13.2 | 39.5 | |
|  | 10 | | 1693 | 6.7 | 40.1 | | 45.0 | 6.1 | 9.7 | | 16.4 | 43.3 | |
| PCa | 11 | | 1736 | 13.0 | 75.6 | | 34.9 | 4.7 | 10.5 | | 7.7 | 20.2 | |
|  | 12 | | 2837 | 6.2 | 6.3 | | 25.9 | 19.4 | 77.2 | | 12.7 | 33.8 | |
|  | 13 | | 4109 | 5.7 | 48.7 | | 32.3 | 16.3 | 23.6 | | < 1.4 | 100.0 | |
|  | 14 | | 3495 | 15.4 | 149.5 | | 20.2 | 12.7 | 64.8 | | 9.6 | 16.7 | |
|  | 15 | | 2872 | 11.7 | 33.4 | | 17.9 | 17.2 | 100.0 | | 5.3 | 68.5 | |
|  | 16 | | 2528 | 16.2 | 33.5 | | 2.3 | 10.5 | 72.8 | | 10.2 | 78.5 | |
|  | 17 | | 1851 | 18.9 | 60.3 | | 87.1 | 8.7 | 69.5 | | 27.2 | 63.0 | |
|  | 18 | | 2479 | 15.2 | 46.3 | | 13.5 | 23.5 | 58.2 | | < 1.4 | 100.0 | |
|  | 19 | | 1940 | 15.3 | 84.5 | | 47.3 | 36.9 | 58.0 | | 4.0 | 72.4 | |
|  | 20 | | 1789 | 20.6 | 73.8 | | 82.8 | 7.3 | 35.5 | | 8.0 | 6.9 | |
|  | 21 | | 2292 | 13.1 | 59.4 | | 77.9 | 9.7 | 50.2 | | 12.8 | 41.6 | |
|  | 22 | | 1503 | 1.0 | 75.0 | | 13.8 | 10.2 | 33.7 | | 11.3 | 100.0 | |
|  | 23 | | 1947 | 6.3 | 68.2 | | 48.3 | 7.5 | 39.2 | | 9.6 | 25.3 | |
|  | 24 | | 2162 | 6.7 | 56.0 | | 38.6 | 6.1 | 38.8 | | < 1.4 | 100.0 | |
|  | 25 | | 5410 | 14.4 | 62.5 | | 54.2 | 9.0 | 18.5 | | 13.6 | 63.4 | |
|  | 26 | | 4152 | 10.2 | 61.7 | | 91.9 | 14.3 | 59.0 | | 22.2 | 24.1 | |
| **Overall results** | | | | | | | | | | | | | |
| Grand average (µM) | | | 2421 | | 53.7 | | | 11.7 | | | 10.6 | | |
| Grand median (µM) | | | 2072 | | 47.9 | | | 10.0 | | | 9.9 | | |
| Grand std. dev. (µM) | | | 952 | | 26.4 | | | 7.0 | | | 6.0 | | |
| Grand std. error (µM) | | | 186.8 | | 5.2 | | | 1.4 | | | 1.2 | | |
| Range (µM) | | | 1213 – 5421 | | 6.3 – 149.5 | | | 2.3 – 36.9 | | | 1.7 – 27.2 | | |
| Avg.envir. Temp. (^o^C) | | | 27.3 ± 1.0 | | 26.4 ± 1.3 | | | 26.3 ± 0.9 | | | 25.9 ± 1.2 | | |
| Avg.envir hum. (%) | | | 52.6 ± 5.0 | | 49.5 ± 7.8 | | | 44.4 ± 9.0 | | | 42.2 ± 10.5 | | |
| Cross-correlation Matrix | | | 1.00 | | 0.17 | | | 0.17 | | | 0.05 | | |
|  |  |  | 0.17 | | 1.00 | | | 0.08 | | | -0.08 | | |
|  |  |  | 0.17 | | 0.08 | | | 1.00 | | | -0.27 | | |
|  |  |  | 0.05 | | -0.08 | | | -0.27 | | | 1.00 | | |
| **non-PCa group** | | | | | | | | | | | | | |
| non-Pca average (µM) | | | 1984 | | 40.2 | | | 10.0 | | | 11.5 | | |
| non-Pca median (µM) | | | 1966 | | 39.8 | | | 9.0 | | | 12.3 | | |
| non-Pca std (µM) | | | 527 | | 11.2 | | | 4.1 | | | 4.3 | | |
| non-Pca std error (µM) | | | 166.6 | | 3.5 | | | 1.3 | | | 1.4 | | |
| Range (µM) | | | 1213 - 3167 | | 21.9 – 67.1 | | | 2.3 – 15.4 | | | 5.1 – 18.8 | | |
| Cross-correlation Matrix | | | 1.00 | | 0.38 | | | 0.17 | | | 0.09 | | |
|  |  |  | 0.38 | | 1.00 | | | 0.18 | | | -0.32 | | |
|  |  |  | 0.17 | | 0.18 | | | 1.00 | | | 0.02 | | |
|  |  |  | 0.09 | | -0.32 | | | 0.02 | | | 1.00 | | |
| **PCa group** | | | | | | | | | | | | | |
| Pca average (µM) | | | 2694 | | 62.2 | | | 13.4 | | | 10.0 | | |
| Pca median (µM) | | | 2386 | | 61.0 | | | 10.4 | | | 9.7 | | |
| Pca std. dev. (µM) | | | 1052 | | 29.5 | | | 7.9 | | | 6.9 | | |
| Pca std error (µM) | | | 263.0 | | 7.4 | | | 2.0 | | | 1.7 | | |
| Range (µM) | | | 1503 - 5410 | | 6.3 – 149.5 | | | 4.7 - 36.9 | | | 1.7 – 27.2 | | |
| Cross-correlation Matrix | | | 1.00 | | -0.02 | | | 0.05 | | | 0.11 | | |
|  |  |  | -0.02 | | 1.00 | | | -0.08 | | | 0.01 | | |
|  |  |  | 0.05 | | -0.08 | | | 1.00 | | | -0.30 | | |
|  |  |  | 0.11 | | 0.01 | | | -0.30 | | | 1.00 | | |
| **Univariate analysis** | | | | | | | | | | | | | |
| Pca/non-Pca (average) | | | 1.36 | | 1.55 | | | 1.34 | | | 0.87 | | |
| Pca/non-Pca (median) | | | 1.21 | | 1.53 | | | 1.15 | | | 0.78 | | |
| t-test (p value) | | | 0.03 | | 0.02 | | | 0.06 | | | 0.27 | | |
|  | | |  | |  | | |  | | |  | | |
| R = 1   | | | R = 0.17   | | | | R = 0.17   | | | | R = 0.05   | | |
| (a) | | | (b) | | | | (c) | | | | (d) | | |
| R = 0.17   | | | R = 1   | | | | R = 0.08   | | | | R = -0.08   | | |
| (e) | | | (f) | | | | (g) | | | | (h) | | |
| R = 0.17   | | | R = 0.08   | | | | R = 1   | | | | R = -0.27   | | |
| (i) | | | (j) | | | | (k) | | | | (l) | | |
| R = 0.05   | | | R = -0.08   | | | | R = -0.27   | | | | R = 1   | | |
| (m) | | | (n) | | | | (o) | | | | (p) | | |

Figure 6. (a)-(p) Scatter plots for all the metabolite combinations. Metabolites were quantified using the platform. Concentrations were normalized to the respective grand average. Blue markers: non-PCa samples. Red markers: PCa samples. Cross-correlation values are referred to the overall dataset.

| R = 0.67   |  |
| --- | --- |
| (a) | (b) |
| R = 0.83   |  |
| (c) | (d) |
| R = 0.80 (vs. MS/MS)    (MS/MS) |  |
| (e) | (f) |
| R = 0.67   |  |
| (g) | (h) |

Figure 7. Normalised metabolite levels measured with this platform compared with reference measurements. (a),(b) LAA (reference: commercial kit ab65347 by Abcam); (c)(d), glutamate (reference: commercial kit ab138883 by Abcam); (e)(f) choline (references: (i) commercial kit ab219944 by Abcam and (ii) ultra-performance liquid chromatography-tandem mass spectrometry; (g)(h) sarcosine (reference: commercial kit ab65338 by Abcam).

### References of supplementary information

[1] D. S. Wishart, “Emerging applications of metabolomics in drug discovery and precision medicine,” *Nat. Rev. Drug Discov.*, vol. 15, no. 7, pp. 473–484, 2016, doi: 10.1038/nrd.2016.32.

[2] G. D. Dakubo, *Cancer Biomarkers in Body Fluids*. Springer International Publishing, 2017.

[3] A. B. Leichtle *et al.*, “Pancreatic carcinoma, pancreatitis, and healthy controls: Metabolite models in a three-class diagnostic dilemma,” *Metabolomics*, vol. 9, no. 3, pp. 677–687, Jun. 2013, doi: 10.1007/s11306-012-0476-7.

[4] L. Wang *et al.*, “1H-NMR based metabonomic profiling of human esophageal cancer tissue,” *Mol. Cancer*, vol. 12, no. 1, p. 25, Apr. 2013, doi: 10.1186/1476-4598-12-25.

[5] J. Shen, L. Yan, S. Liu, C. B. Ambrosone, and H. Zhao, “Plasma metabolomic profiles in breast cancer patients and healthy controls: By race and tumor receptor subtypes,” *Transl. Oncol.*, vol. 6, no. 6, pp. 757–765, 2013, doi: 10.1593/tlo.13619.

[6] C. Oakman *et al.*, “Identification of a serum-detectable metabolomic fingerprint potentially correlated with the presence of micrometastatic disease in early breast cancer patients at varying risks of disease relapse by traditional prognostic methods,” *Ann. Oncol.*, vol. 22, no. 6, pp. 1295–1301, 2011, doi: 10.1093/annonc/mdq606.

[7] Y. Li, X. Song, X. Zhao, L. Zou, and G. Xu, “Serum metabolic profiling study of lung cancer using ultra high performance liquid chromatography/quadrupole time-of-flight mass spectrometry,” *J. Chromatogr. B Anal. Technol. Biomed. Life Sci.*, vol. 966, pp. 147–153, Sep. 2014, doi: 10.1016/j.jchromb.2014.04.047.

[8] W. Lv and T. Yang, “Identification of possible biomarkers for breast cancer from free fatty acid profiles determined by GC-MS and multivariate statistical analysis,” *Clin. Biochem.*, vol. 45, no. 1–2, pp. 127–133, Jan. 2012, doi: 10.1016/j.clinbiochem.2011.10.011.

[9] L. Tenori *et al.*, “Serum metabolomic profiles evaluated after surgery may identify patients with oestrogen receptor negative early breast cancer at increased risk of disease recurrence. Results from a retrospective study,” *Mol. Oncol.*, vol. 9, no. 1, pp. 128–139, Jan. 2015, doi: 10.1016/j.molonc.2014.07.012.

[10] J. Budczies *et al.*, “Comparative metabolomics of estrogen receptor positive and estrogen receptor negative breast cancer: Alterations in glutamine and beta-alanine metabolism,” *J. Proteomics*, vol. 94, pp. 279–288, Dec. 2013, doi: 10.1016/j.jprot.2013.10.002.

[11] N. Lefort *et al.*, “1H NMR metabolomics analysis of the effect of dichloroacetate and allopurinol on breast cancers,” *J. Pharm. Biomed. Anal.*, vol. 93, pp. 77–85, 2014, doi: 10.1016/j.jpba.2013.08.017.

[12] A. S. Krall, S. Xu, T. G. Graeber, D. Braas, and H. R. Christofk, “Asparagine promotes cancer cell proliferation through use as an amino acid exchange factor,” *Nat. Commun.*, vol. 7, pp. 1–13, Apr. 2016, doi: 10.1038/ncomms11457.

[13] S. R. V. Knott *et al.*, “Asparagine bioavailability governs metastasis in a model of breast cancer,” *Nature*, vol. 554, no. 7692, pp. 378–381, Feb. 2018, doi: 10.1038/nature25465.

[14] H. Nam, B. C. Chung, Y. Kim, K. Y. Lee, and D. Lee, “Combining tissue transcriptomics and urine metabolomics for breast cancer biomarker identification,” *Bioinformatics*, vol. 25, no. 23, pp. 3151–3157, Sep. 2009, doi: 10.1093/bioinformatics/btp558.

[15] A. Zhang, H. Sun, G. Yan, P. Wang, Y. Han, and X. Wang, “Metabolomics in diagnosis and biomarker discovery of colorectal cancer,” *Cancer Lett.*, vol. 345, no. 1, pp. 17–20, Apr. 2014, doi: 10.1016/j.canlet.2013.11.011.

[16] A. B. Leichtle *et al.*, “Serum amino acid profiles and their alterations in colorectal cancer,” *Metabolomics*, vol. 8, no. 4, pp. 643–653, Aug. 2012, doi: 10.1007/s11306-011-0357-5.

[17] H. Wang, V. K. Tso, C. M. Slupsky, and R. N. Fedorak, “Metabolomics and detection of colorectal cancer in humans: A systematic review,” *Future Oncology*, vol. 6, no. 9. pp. 1395–1406, Sep. 2010, doi: 10.2217/fon.10.107.

[18] Y. Qiu *et al.*, “Serum metabolite profiling of human colorectal cancer using GC-TOFMS and UPLC-QTOFMS,” *J. Proteome Res.*, vol. 8, no. 10, pp. 4844–4850, 2009, doi: 10.1021/pr9004162.

[19] A. Huang, D. Fuchs, B. Widner, C. Glover, D. C. Henderson, and T. G. Allen-Mersh, “Tryptophan and quality of life in colorectal cancer,” in *Advances in Experimental Medicine and Biology*, 2003, vol. 527, pp. 353–358, doi: 10.1007/978-1-4615-0135-0_39.

[20] S. A. Ritchie *et al.*, “Reduced levels of hydroxylated, polyunsaturated ultra long-chain fatty acids in the serum of colorectal cancer patients: Implications for early screening and detection,” *BMC Med.*, vol. 8, Feb. 2010, doi: 10.1186/1741-7015-8-13.

[21] B. Jiménez *et al.*, “1H HR-MAS NMR spectroscopy of tumor-induced local metabolic ‘field-effects’ enables colorectal cancer staging and prognostication,” *J. Proteome Res.*, vol. 12, no. 2, pp. 959–968, Feb. 2013, doi: 10.1021/pr3010106.

[22] K. Raina, K. Ravichandran, S. Rajamanickam, K. M. Huber, N. J. Serkova, and R. Agarwal, “Inositol hexaphosphate inhibits tumor growth, vascularity, and metabolism in TRAMP mice: A multiparametric magnetic resonance study,” *Cancer Prev. Res.*, vol. 6, no. 1, pp. 40–50, Jan. 2013, doi: 10.1158/1940-6207.CAPR-12-0387.

[23] G. F. Giskeødegård *et al.*, “Spermine and Citrate as Metabolic Biomarkers for Assessing Prostate Cancer Aggressiveness,” *PLoS One*, vol. 8, no. 4, Apr. 2013, doi: 10.1371/journal.pone.0062375.

[24] J. E. McDunn *et al.*, “Metabolomic signatures of aggressive prostate cancer,” *Prostate*, vol. 73, no. 14, pp. 1547–1560, Oct. 2013, doi: 10.1002/pros.22704.

[25] O. F. Bathe *et al.*, “Feasibility of identifying pancreatic cancer based on serum metabolomics,” *Cancer Epidemiol. Biomarkers Prev.*, vol. 20, no. 1, pp. 140–147, 2011, doi: 10.1158/1055-9965.EPI-10-0712.

[26] D. O. Yang, J. Xu, H. Huang, and Z. Chen, “Metabolomic profiling of serum from human pancreatic cancer patients Using 1 H NMR spectroscopy and principal component analysis,” *Appl. Biochem. Biotechnol.*, vol. 165, no. 1, pp. 148–154, 2011, doi: 10.1007/s12010-011-9240-0.

[27] L. Zhang *et al.*, “Distinguishing pancreatic cancer from chronic pancreatitis and healthy individuals by 1H nuclear magnetic resonance-based metabonomic profiles,” *Clin. Biochem.*, vol. 45, no. 13–14, pp. 1064–1069, Sep. 2012, doi: 10.1016/j.clinbiochem.2012.05.012.

[28] S. Chen *et al.*, “Pseudotargeted metabolomics method and its application in serum biomarker discovery for hepatocellular carcinoma based on ultra high-performance liquid chromatography/triple quadrupole mass spectrometry,” *Anal. Chem.*, vol. 85, no. 17, pp. 8326–8333, 2013, doi: 10.1021/ac4016787.

[29] S. Urayama, W. Zou, K. Brooks, and V. Tolstikov, “Comprehensive mass spectrometry based metabolic profiling of blood plasma reveals potent discriminatory classifiers of pancreatic cancer,” *Rapid Commun. Mass Spectrom.*, vol. 24, no. 5, pp. 613–620, Mar. 2010, doi: 10.1002/rcm.4420.

[30] J. F. Xiao *et al.*, “LC-MS based serum metabolomics for identification of hepatocellular carcinoma biomarkers in Egyptian cohort,” *J. Proteome Res.*, vol. 11, no. 12, pp. 5914–5923, Dec. 2012, doi: 10.1021/pr300673x.

[31] S. A. Ritchie *et al.*, “Metabolic system alterations in pancreatic cancer patient serum: Potential for early detection,” *BMC Cancer*, vol. 13, Sep. 2013, doi: 10.1186/1471-2407-13-416.

[32] P. Tripathi *et al.*, “Delineating metabolic signatures of head and neck squamous cell carcinoma: Phospholipase A2, a potential therapeutic target,” *Int. J. Biochem. Cell Biol.*, vol. 44, no. 11, pp. 1852–1861, Nov. 2012, doi: 10.1016/j.biocel.2012.06.025.

[33] J. D. Clarke *et al.*, “Characterization of hepatocellular carcinoma related genes and metabolites in human nonalcoholic fatty liver disease,” *Dig. Dis. Sci.*, vol. 59, no. 2, pp. 365–374, Feb. 2014, doi: 10.1007/s10620-013-2873-9.

[34] H. Wen *et al.*, “A new NMR-based metabolomics approach for the diagnosis of biliary tract cancer,” *J. Hepatol.*, vol. 52, no. 2, pp. 228–233, Feb. 2010, doi: 10.1016/j.jhep.2009.11.002.

[35] X. H. He *et al.*, “Metabonomic studies of pancreatic cancer response to radiotherapy in a mouse xenograft model using magnetic resonance spectroscopy and principal components analysis,” *World J. Gastroenterol.*, vol. 19, no. 26, pp. 4200–4208, Jul. 2013, doi: 10.3748/wjg.v19.i26.4200.

[36] T. Zhang *et al.*, “Discrimination between malignant and benign ovarian tumors by plasma metabolomic profiling using ultra performance liquid chromatography/mass spectrometry,” *Clin. Chim. Acta*, vol. 413, no. 9–10, pp. 861–868, May 2012, doi: 10.1016/j.cca.2012.01.026.

[37] W. Struck, D. Siluk, A. Yumba-Mpanga, M. Markuszewski, R. Kaliszan, and M. J. Markuszewski, “Liquid chromatography tandem mass spectrometry study of urinary nucleosides as potential cancer markers,” *J. Chromatogr. A*, vol. 1283, pp. 122–131, Mar. 2013, doi: 10.1016/j.chroma.2013.01.111.

[38] J. V. Alberice *et al.*, “Searching for urine biomarkers of bladder cancer recurrence using a liquid chromatography-mass spectrometry and capillary electrophoresis-mass spectrometry metabolomics approach,” *J. Chromatogr. A*, vol. 1318, pp. 163–170, Nov. 2013, doi: 10.1016/j.chroma.2013.10.002.

[39] P. Tripathi *et al.*, “HR-MAS NMR tissue metabolomic signatures cross-validated by mass spectrometry distinguish bladder cancer from benign disease,” *J. Proteome Res.*, vol. 12, no. 7, pp. 3519–3528, Jul. 2013, doi: 10.1021/pr4004135.

[40] J. Huang *et al.*, “Serum metabolomic profiling of prostate cancer risk in the prostate, lung, colorectal, and ovarian cancer screening trial,” *Br. J. Cancer*, vol. 115, no. 9, pp. 1087–1095, Oct. 2016, doi: 10.1038/bjc.2016.305.

[41] M. Kdadra, S. Höckner, H. Leung, W. Kremer, and E. Schiffer, “Metabolomics biomarkers of prostate cancer: A systematic review,” *Diagnostics*, vol. 9, no. 1, pp. 1–44, Feb. 2019, doi: 10.3390/diagnostics9010021.

[42] R. S. Kelly, M. G. V. Heiden, E. Giovannucci, and L. A. Mucci, “Metabolomic biomarkers of prostate cancer: Prediction, diagnosis, progression, prognosis, and recurrence,” *Cancer Epidemiol. Biomarkers Prev.*, vol. 25, no. 6, pp. 887–906, Jun. 2016, doi: 10.1158/1055-9965.EPI-15-1223.

[43] G. F. Giskeødegård *et al.*, “Metabolic markers in blood can separate prostate cancer from benign prostatic hyperplasia,” *Br. J. Cancer*, vol. 113, no. 12, pp. 1712–1719, Dec. 2015, doi: 10.1038/bjc.2015.411.

[44] M. Johansson *et al.*, “One-carbon metabolism and prostate cancer risk: Prospective investigation of seven circulating B vitamins and metabolites,” *Cancer Epidemiol. Biomarkers Prev.*, vol. 18, no. 5, pp. 1538–1543, May 2009, doi: 10.1158/1055-9965.EPI-08-1193.

[45] C. Myers *et al.*, “Suramin: A novel growth factor antagonist with activity in hormone- refractory metastatic prostate cancer,” *J. Clin. Oncol.*, vol. 10, no. 6, pp. 881–889, 1992, doi: 10.1200/JCO.1992.10.6.881.

[46] S. J. Weinstein, K. Mackrain, R. Z. Stolzenberg-Solomon, J. Selhub, J. Virtamo, and D. Albanes, “Serum creatinine and prostate cancer risk in a prospective study,” *Cancer Epidemiol. Biomarkers Prev.*, vol. 18, no. 10, pp. 2643–2649, Oct. 2009, doi: 10.1158/1055-9965.EPI-09-0322.

[47] P. M. Quilty *et al.*, “A comparison of the palliative effects of strontium-89 and external beam radiotherapy in metastatic prostate cancer,” *Radiother. Oncol.*, vol. 31, no. 1, pp. 33–40, 1994, doi: 10.1016/0167-8140(94)90411-1.

[48] A. C. Ogilvie, J. C. Van Der Mijn, M. J. Kuiper, C. E. H. Siegert, A. E. Wassenaar, and C. J. M. Van Noesel, “Lactic Acidosis in Prostate Cancer: Consider the Warburg Effect Keywords Mutation · p53 mutations · PIK3CA mutations · PTEN mutations · IDH1 mutations · Prostate cancer · Lactic acidosis · Warburg effect · Metabolic reprogramming · Case report,” *Case Rep Oncol*, vol. 10, pp. 1085–1091, 2017, doi: 10.1159/000485242.

[49] K. Fabyan, A. Holtzclaw, and J. Sherner, “PROSTATE LACTATE: A CASE OF SEVERE LACTIC ACIDOSIS WITH AN INTERESTING MECHANISM OF ACTION,” *Chest*, vol. 156, no. 4, p. A2105, Oct. 2019, doi: 10.1016/j.chest.2019.08.2050.

[50] B. J. Trock, “Application of metabolomics to prostate cancer,” *Urologic Oncology: Seminars and Original Investigations*, vol. 29, no. 5. pp. 572–581, Sep. 2011, doi: 10.1016/j.urolonc.2011.08.002.

[51] A. Sreekumar *et al.*, “Metabolomic profiles delineate potential role for sarcosine in prostate cancer progression,” *Nature*, vol. 457, no. 7231, pp. 910–914, Feb. 2009, doi: 10.1038/nature07762.

[52] E. Thysell *et al.*, “Metabolomic characterization of human prostate cancer bone metastases reveals increased levels of cholesterol,” *PLoS One*, vol. 5, no. 12, 2010, doi: 10.1371/journal.pone.0014175.

[53] J. W. Locasale, “Serine, glycine and one-carbon units: Cancer metabolism in full circle,” *Nature Reviews Cancer*, vol. 13, no. 8. pp. 572–583, Aug. 2013, doi: 10.1038/nrc3557.

[54] X. Zhang *et al.*, “Metabolic signatures of esophageal cancer: NMR-based metabolomics and UHPLC-based focused metabolomics of blood serum,” *Biochim. Biophys. Acta - Mol. Basis Dis.*, vol. 1832, no. 8, pp. 1207–1216, Aug. 2013, doi: 10.1016/j.bbadis.2013.03.009.

[55] J. Zhang *et al.*, “Esophageal Cancer Metabolite Biomarkers Detected by LC-MS and NMR Methods,” *PLoS One*, vol. 7, no. 1, p. e30181, Jan. 2012, doi: 10.1371/journal.pone.0030181.

[56] T. Wiggins, S. Kumar, S. R. Markar, S. Antonowicz, and G. B. Hanna, “Tyrosine, phenylalanine, and tryptophan in gastroesophageal malignancy: A systematic review,” *Cancer Epidemiol. Biomarkers Prev.*, vol. 24, no. 1, pp. 32–38, Jan. 2015, doi: 10.1158/1055-9965.EPI-14-0980.

[57] Q. Y. *et al.*, “A distinct metabolic signature of human colorectal cancer with prognostic potential,” *Clin. Cancer Res.*, vol. 20, no. 8, pp. 2136–2146, 2014, doi: 10.1158/1078-0432.CCR-13-1939 LK - http://resolver.ebscohost.com/openurl?sid=EMBASE&issn=15573265&id=doi:10.1158%2F1078-0432.CCR-13-1939&atitle=A+distinct+metabolic+signature+of+human+colorectal+cancer+with+prognostic+potential&stitle=Clin.+Cancer+Res.&title=Clinical+Cancer+Research&volume=20&issue=8&spage=2136&epage=2146&aulast=Qiu&aufirst=Yunping&auinit=Y.&aufull=Qiu+Y.&coden=CCREF&isbn=&pages=2136-2146&date=2014&auinit1=Y&auinitm=.

[58] K. Yonezawa *et al.*, “Serum and tissue metabolomics of head and neck cancer,” *Cancer Genomics and Proteomics*, vol. 10, no. 5, pp. 233–238, 2013.

[59] T. Wen *et al.*, “Exploratory investigation of plasma metabolomics in human lung adenocarcinoma,” *Mol. Biosyst.*, vol. 9, no. 9, pp. 2370–2378, 2013, doi: 10.1039/c3mb70138g.

[60] Y. Guo *et al.*, “Probing gender-specific lipid metabolites and diagnostic biomarkers for lung cancer using Fourier transform ion cyclotron resonance mass spectrometry,” *Clin. Chim. Acta*, vol. 414, pp. 135–141, Dec. 2012, doi: 10.1016/j.cca.2012.08.010.

[61] L. Gao, Z. Wen, C. Wu, T. Wen, and C. Ong, “Metabolic Profiling of Plasma from Benign and Malignant Pulmonary Nodules Patients Using Mass Spectrometry-Based Metabolomics,” *Metabolites*, vol. 3, no. 3, pp. 539–551, Jul. 2013, doi: 10.3390/metabo3030539.

[62] M. Serizawa *et al.*, “Identification of metabolic signatures associated with erlotinib resistance of non-small cell lung cancer cells.,” *Anticancer Res.*, vol. 34, no. 6, pp. 2779–87, Jun. 2014, Accessed: Jan. 20, 2020. [Online]. Available: http://www.ncbi.nlm.nih.gov/pubmed/24922639.

[63] A. P. J. Van Den Heuvel, J. Jing, R. F. Wooster, and K. E. Bachman, “Analysis of glutamine dependency in non-small cell lung cancer: GLS1 splice variant GAC is essential for cancer cell growth,” *Cancer Biol. Ther.*, vol. 13, no. 12, pp. 1185–1194, Oct. 2012, doi: 10.4161/cbt.21348.

[64] S. Nakamizo *et al.*, “GC/MS-based metabolomic analysis of cerebrospinal fluid (CSF) from glioma patients,” *J. Neurooncol.*, vol. 113, no. 1, pp. 65–74, May 2013, doi: 10.1007/s11060-013-1090-x.

[65] S. G. Musharraf, A. J. Siddiqui, T. Shamsi, M. I. Choudhary, and A. U. Rahman, “Serum metabonomics of acute leukemia using nuclear magnetic resonance spectroscopy,” *Sci. Rep.*, vol. 6, Aug. 2016, doi: 10.1038/srep30693.

[66] D. A. MacIntyre *et al.*, “Serum metabolome analysis by 1H-NMR reveals differences between chronic lymphocytic leukaemia molecular subgroups,” *Leukemia*, vol. 24, no. 4, pp. 788–797, 2010, doi: 10.1038/leu.2009.295.

[67] B. Tan *et al.*, “Metabonomics identifies serum metabolite markers of colorectal cancer,” *J. Proteome Res.*, vol. 12, no. 6, pp. 3000–3009, 2013, doi: 10.1021/pr400337b.

[68] B. S. Li *et al.*, “The downregulation of asparagine synthetase expression can increase the sensitivity of cells resistant to L-asparaginase [13],” *Leukemia*, vol. 20, no. 12. Nature Publishing Group, pp. 2199–2201, 2006, doi: 10.1038/sj.leu.2404423.

[69] S. Holst *et al.*, “Investigations on aberrant glycosylation of glycosphingolipids in colorectal cancer tissues using liquid chromatography and matrix-assisted laser desorption time-of- flight mass spectrometry (MALDI-TOF-MS),” *Mol. Cell. Proteomics*, vol. 12, no. 11, pp. 3081–3093, Nov. 2013, doi: 10.1074/mcp.M113.030387.

[70] “Enzyme Database - BRENDA.” https://www.brenda-enzymes.org/index.php (accessed Jan. 22, 2020).

[71] M. A. Al-Rawhani *et al.*, “Multimodal Integrated Sensor Platform for Rapid Biomarker Detection.,” *IEEE Trans. Biomed. Eng.*, Jun. 2019, doi: 10.1109/TBME.2019.2919192.

[72] P. Jóźwiak, E. Forma, M. Bryś, and A. Krześlak, “O-GlcNAcylation and metabolic reprograming in cancer,” *Frontiers in Endocrinology*, vol. 5, no. SEP. Frontiers Media S.A., 2014, doi: 10.3389/fendo.2014.00145.

[73] B. Kalyanaraman, “Teaching the basics of cancer metabolism: Developing antitumor strategies by exploiting the differences between normal and cancer cell metabolism,” *Redox Biology*, vol. 12. Elsevier B.V., pp. 833–842, Aug. 01, 2017, doi: 10.1016/j.redox.2017.04.018.

[74] A. Vazquez, J. J. Kamphorst, E. K. Markert, Z. T. Schug, S. Tardito, and E. Gottlieb, “Cancer metabolism at a glance,” *J. Cell Sci.*, vol. 129, no. 18, pp. 3367–3373, 2016, doi: 10.1242/jcs.181016.

[75] L. M. Sanders and S. H. Zeisel, “Choline: Dietary requirements and role in brain development,” *Nutr. Today*, vol. 42, no. 4, pp. 181–186, 2007, doi: 10.1097/01.NT.0000286155.55343.fa.

[76] A. R. Meyer and M. A. Gorin, “First point-of-care PSA test for prostate cancer detection,” *Nat. Rev. Urol.*, vol. 16, no. 6, pp. 332–333, 2019, doi: 10.1038/s41585-019-0179-1.

[77] S. Koochekpour *et al.*, “Serum glutamate levels correlate with gleason score and glutamate blockade decreases proliferation, migration, and invasion and induces apoptosis in prostate cancer cells,” *Clin. Cancer Res.*, vol. 18, no. 21, pp. 5888–5901, Nov. 2012, doi: 10.1158/1078-0432.CCR-12-1308.

[78] L. C. Soliman, Y. Hui, A. K. Hewavitharana, and D. D. Y. Chen, “Monitoring potential prostate cancer biomarkers in urine by capillary electrophoresis-tandem mass spectrometry,” *J. Chromatogr. A*, vol. 1267, pp. 162–169, Dec. 2012, doi: 10.1016/j.chroma.2012.07.021.

[79] “NUCLEO-F334R8 - STM32 Nucleo-64 development board with STM32F334R8 MCU, supports Arduino and ST morpho connectivity - STMicroelectronics.” https://www.st.com/en/evaluation-tools/nucleo-f334r8.html (accessed Feb. 05, 2020).

[80] “Mbed Compiler Workspace Management.” https://ide.mbed.com/compiler/#nav:/; (accessed Feb. 05, 2020).

[81] M. Bigas, E. Cabruja, J. Forest, and J. Salvi, “Review of CMOS image sensors,” 2005, doi: 10.1016/j.mejo.2005.07.002.

[82] C. Accarino *et al.*, “Noise characteristics with CMOS sensor array scaling,” *Meas. J. Int. Meas. Confed.*, vol. 152, Feb. 2020, doi: 10.1016/j.measurement.2019.107325.

[83] N. Psychogios *et al.*, “The human serum metabolome,” *PLoS One*, vol. 6, no. 2, 2011, doi: 10.1371/journal.pone.0016957.
